# PEA3 Transcription Factors, Role in Invasion, Proliferation and Radioresistance of Glioblastoma Stem Cells

**DOI:** 10.1111/jcmm.70533

**Published:** 2025-04-24

**Authors:** Yvan Nicaise, Caroline Delmas, Elizabeth Cohen‐Jonathan‐Moyal, Catherine Seva

**Affiliations:** ^1^ Centre de Recherche en Cancérologie de Toulouse (CRCT) INSERM U1037, Université Toulouse III‐Paul Sabatier Toulouse France; ^2^ IUCT‐Oncopole, Institut Claudius Regaud Toulouse France

**Keywords:** glioblastoma stem cells, invasion, PEA3 transcription factors, radioresistance

## Abstract

The presence of glioblastoma stem cells (GSCs), known for their high invasiveness and resistance to radiation, is one of the reasons for systematic recurrence. It is therefore important to understand the resistance mechanisms of these cells to optimise therapies. We focused on the PEA3 family of transcription factors, ETV1, ETV4 and ETV5, in patient‐derived GSCs. We demonstrate that ETV1 is over‐expressed in high invasive GSCs. In 3D invasion assays, inhibiting ETV1 expression using specific siRNAs significantly reduces cell invasion. Furthermore, we show a significant correlation between ETV1 and ZEB1, a major driver of invasion. Blocking ETV1 decreases ZEB1 expression in GSCs. The study also demonstrates the essential role of ETV1, ETV4 and ETV5 in the radioresistance of GSCs and their ability to form neurospheres. Using specific siRNAs to inhibit the expression of these transcription factors led to an increased sensitivity of GSCs to radiation and a decrease in both the number and size of neurospheres. These findings suggest that PEA3 transcription factors play a major role in GSCs aggressiveness by regulating invasion, radioresistance and the ability to form neurospheres.

**Trial Registration:** Registry and the Registration N° of the study/trial: 12TETE01, ID‐RCB No. 2012‐A00585‐38, approval Date: May 7, 2012

## 1 Introduction

Glioblastoma (GBM) is the most aggressive brain tumour in adults. GBM cells have a high invasive potential, making surgical removal challenging. Additionally, GBM is particularly resistant to post‐operative adjuvant therapies. The majority of GBM cases relapse despite radio‐ and chemotherapy, and the median survival remains around 15 months [1]. The presence of Glioblastoma Stem Cells (GSCs) within the tumour, known for their high tumorigenic capacity in vivo, their ability to self‐renew and their pluripotent aptitude is one of the causes of this high recurrence. GSCs have a high potential to penetrate surrounding normal brain and are extremely chemo‐radio‐resistant [2, 3]. The mechanisms regulating GSCs invasive capacity or their resistance to therapies are not fully understood, and there is currently no clinical treatment to specifically target GSCs. Consequently, deciphering the molecular mechanisms underlying GSCs resistance and invasiveness is a necessity to develop new effective therapies for GBM.

In the present paper, we focused on the PEA3 subfamily of transcription factors that belongs to the large family of E26 transformation‐specific (ETS) transcription factors. It includes three members: ETV1, ETV4 and ETV5. They are recognised as pro‐oncogenic transcription factors with an important role in tumour progression in many cancers. They contribute to cell proliferation and tumour growth as well as invasion processes and metastasis formation, mainly in gastrointestinal stromal tumours (GISTs), colorectal, gastric, pancreatic, Breast, prostate and hepatocellular carcinoma [4, 5]. In addition, the PEA3 transcription factors have been also involved in resistance to chemotherapies in different cancers [6, 7, 8, 9, 10]. In contrast, their role in radioresistance has never been reported. Unlike the extensive bibliography that exists on other cancers, very few data have been published on the expression and the role of PEA3 transcription factors in GBM. A poor survival prognosis has recently been associated with a high expression of ETV4 or ETV5 for patients with GBM [11, 12]. The same authors also report a role for ETV4 in the activation of survival pathways and conversely an induction of cell apoptosis when this transcription factor is inhibited [12]. Several papers also describe different molecular mechanisms regulating the expression of the PEA3 transcription factors in GBM. Lin et al. show the regulation of ETV5 expression by the Insulin‐like Growth Factor‐Binding Protein 5 (IGFBP5), a ligand of the ROR1 receptor, which promotes GBM invasion [13]. ETV1, ETV4 and ETV5 are known target genes of the transcriptional repressor Capicua (CIC). Loss of function or expression of CIC in numerous tumours can lead to overexpression of PEA3 transcription factors. However, in GBM conflicting data have been reported. The work of Bunda et al. [14] suggests that Src‐family kinases inhibit CIC functions by phosphorylation and increase ETV1 and ETV5 expression. According to these results, another study has shown that gliomas expressing an inactive mutant of CIC display an up‐regulation of ETV1, ET4 and ETV5 [15]. On the contrary, Park's study reports that CIC modulation does not affect the expression of ETV1, ETV4 and ETV5 in GBM cells [16]. Finally, SRSF3, a major splicing factor that promotes GBM tumorigenicity, also controls ETV1 expression [17].

The expression and the role of PEA3 transcription factor has been little studied in GSCs. More particularly, the role of these factors in GSCs resistance and invasiveness has never been reported. In the present study we analysed the expression of these transcription factors and their function in GSCs. We report that ETV1 is overexpressed in high invasive GSCs. We also demonstrate that targeting ETV1 represses cell invasion. In addition, we also demonstrate that blocking the expression of ETV1, ETV4 or ETV5 decreases cell proliferation, neurosphere formation and increases radiosensitivity of GSCs.

## 2 Materials and Methods

### 2.1 GSCs Derived From GBM Biopsy Specimens

GBM biopsies were acquired from the Department of Neurosurgery at Toulouse University Hospital. This clinical study, led by Professor E. Cohen‐Jonathan‐Moyal, received approval from the Human Research Ethics Committee (Ethics Code 12TETE01, ID‐RCB No. 2012‐A00585‐38, Approval Date: May 7, 2012). All patients provided written informed consent. The primary neurospheres of GSCs were obtained from GBM samples following the method described by Avril [18] and cultured in DMEM‐F12 (GIBCO) supplemented with B27 and N2 (Life‐Technologies), FGF‐2, and EGF (Peprotech). The GSCs used in this study have been previously characterised and possess self‐renewal capacity, overexpression of stem cell markers, the ability to differentiate into neural lineages, and the capability to form tumours in vivo [19, 20, 21].

### 2.2 RNA Sequencing

We used the RNeasy kit (Qiagen) to extract RNA from GSCs, and then carried out the quantification using the RNA Qubit Broad Range Kit (Thermo Fisher Scientific). We verified the purity using the NanoDrop ND‐100 (Thermo Fisher Scientific). We checked the RNA quality using a fragment analyser (Agilent Technology) to obtain RIN (RNA integrity number) and verify the absence of genomic DNA. For library preparation, 400 ng of total RNA was used. NGS‐based RNA sequencing was performed on a Nextseq 550 instrument using Illumina Stranded Total RNA Prep, and ligation with Ribo‐Zero Plus Kit (Illumina) following the manufacturer protocol (Illumina, San Diego, CA, USA). The RNAseq data presented in this study are openly available in the SRA database under the reference PRJNA1020743.

### 2.3 Bioinformatic Analysis

The mRNA levels of ETV1, ETV4, and ETV5 were analysed in non‐tumour brain samples, GBM, low‐grade glioma (LGG) and GSCs. Data for non‐tumour brain samples, GBM samples or LGG were downloaded from the TCGA or Rembrandt databases, while the data for GSCs were obtained from RNAseq analysis performed on GSCs derived from 13 human GBM biopsy specimens. To compare the expression levels, all the data were normalised using GAPDH expression. Unpaired *t*‐tests were conducted to compare two groups. One‐way Anova tests were performed for multiple comparisons.

Kaplan–Meier estimator survival analysis was performed to estimate the probability of overall survival between patients with ETV1, ETV4 or ETV5 high and low expression. The analysis was performed from TCGA or Rembrandt databases using the Giovis website (http://gliovis.bioinfo.cnio.es). Wilcoxon tests were used in this analysis.

The differentially expressed genes between GSCs expressing high or low levels of ETV1 were determined from the RNAseq dataset of the 13 patient samples by comparing for each gene the average expression of the ETV1‐high group to that of the ETV1‐low group (fold change cut off ≥ 2.0 and *p* < 0.05). The results are visualised with a volcano plot performed with Srplot, (http://www.bioinformatics.com.cn/srplot), an online platform for data analysis and visualisation. We also used the Gliovis website (http://gliovis.bioinfo.cnio.es) to analyse from GBM of the TCGA and Rembrandt databases the genes differentially expressed between GBM with a high or low ETV1 expression (fold change cut off ≥ 2.0 and *p* < 0.05).

The Metascape platform (https://metascape.org), a gene annotation and analysis resource, was used to conduct gene ontology analysis on the genes that showed significant up‐regulation in GSCs expressing high levels of ETV1. To visualise the main pathways, an enrichment bubble plot was generated using Srplot (http://www.bioinformatics.com.cn/srplot), an online platform for data analysis and visualisation.

The correlations between ETV1 expression and PTPRZ1 or ZEB1 in GBM samples of the TCGA or Rembrandt databases were determined through co‐expression analysis using the Gliovis website (http://gliovis.bioinfo.cnio.es). In GSCs, the correlations between ETV1 expression and PTPRZ1 or ZEB1 obtained by RNAseq were determined with the Excel software. The correlations between ETV1, ETV4 or ETV5 expression in GSCs and invasion data were also analysed with the Excel software. Pearson correlation coefficients and their associated *p*‐values were used to evaluate the link between biomarkers or biomarkers and invasion level.

### 2.4 Western‐Blot Analysis

Identical levels of proteins were separated by SDS‐PAGE and analysed by western‐blot with the indicated antibodies: ETV1, ETV4, ETV5 (Invitrogen), actin (Millipore), ZEB1 (Cell Signalling).

### 2.5 3D Tumour Spheroid Invasion Assay

The 3D tumour spheroid invasion assays were conducted according to the method described by Vinci M. et al. [22]. The spheroids were transfected with either specific siRNA or a scramble control using Lipofectamine RNAi Max Invitrogen. Images were taken at T0 and T24h using a Nikon microscope and the Nikon software NIS Elements. The area of the spheres and the area occupied by the invading cells were quantified using the ImageJ software.

### 2.6 SiRNA Transfection, RNA Extraction, Reverse Transcription and Real‐Time PCR

Lipofectamine RNAi Max from Invitrogen was used for transfection following the manufacturer's protocol. 48 h post‐transfection, total RNA was extracted using the RNeasy RNA isolation Kit from Qiagen, and reverse transcription was carried out with the Prime Script RT Reagent kit from TAKARA. Real‐time PCR was performed using the ABI‐Stepone+ system from Applied Biosystems, with GAPDH used for normalisation. The following siRNA were used: si‐ETV1(1), si‐ETV1(2) (respectively S4855 and S223504 from Ambion), si‐ETV4(1), si‐ETV4(2) (respectively SI00030954, SI00030961, from Qiagen), si‐ETV5(1), si‐ETV5(2) (respectively SI03019394, SI04274396, from Qiagen) and si‐ZEB1 (SI04272492 from Qiagen).

### 2.7 Neurosphere‐Forming Analysis

Cells were transfected with specific siRNAs or a scramble control and then seeded in 96‐well plates at a density of 500 cells per well, with 12 wells per condition. After 48 h, the cells were either irradiated or not with increasing doses of x‐rays ranging from 0 to 6 Gy (SmART+ irradiator, Precision X‐ray Inc., Madison, USA). After 8–10 days, the number of neurospheres per well was counted under a microscope. The surviving fraction (SF) was calculated as previously described, survival = PE × Gy/PE 0 gy ×100 (PE = plating efficiency). The spheres size was measured using the ImageJ software.

## 3 Results

### 3.1 Expression Levels of PEA3 Transcription Factors in GBM and GSCs

**FIGURE 1:**
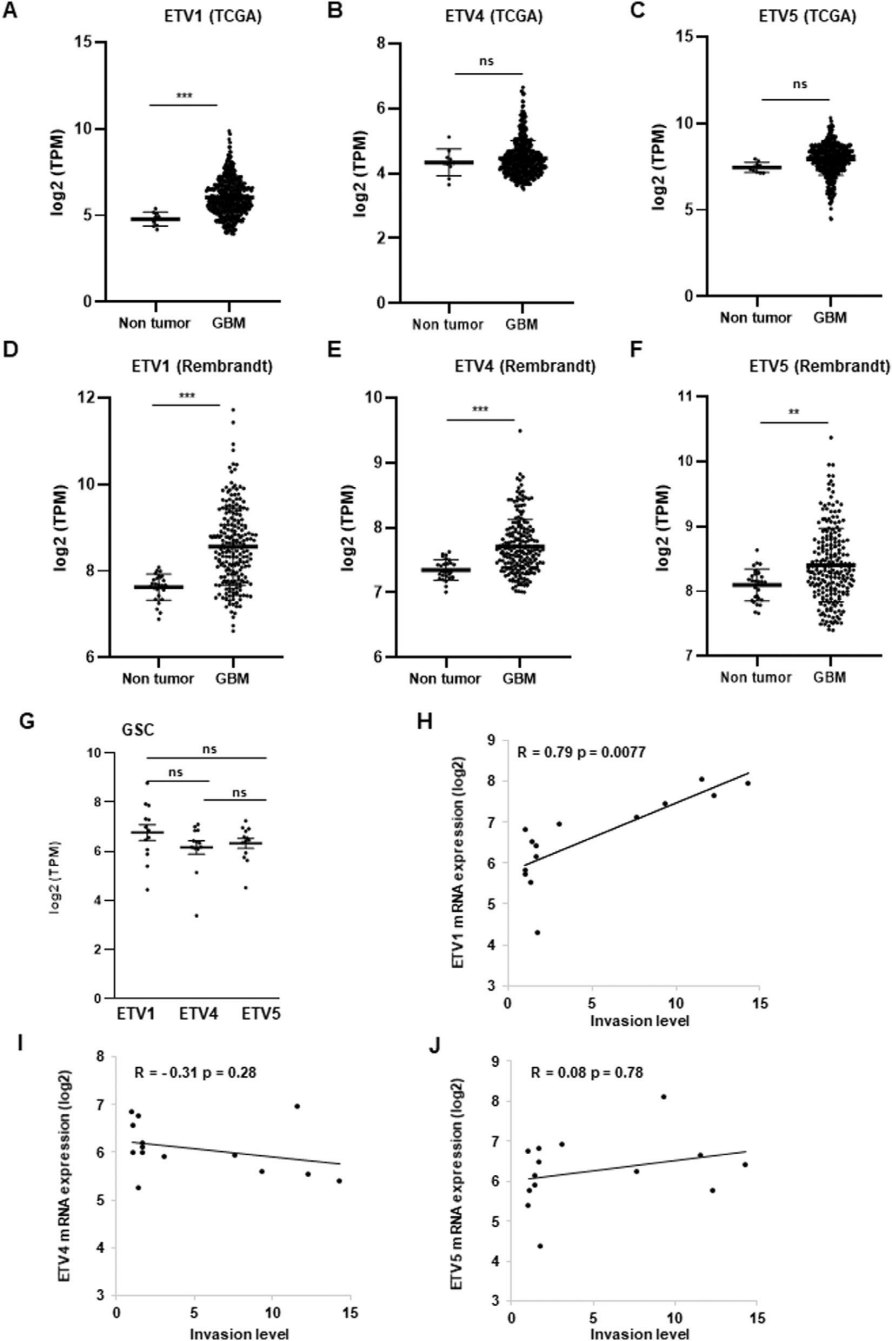
Expression of PEA3 transcription factors in GBM and GSCs and correlations with invasion of GSCs. (A–F) the levels of ETV1, ETV4 and ETV5 mRNA in GBM from the TCGA database (*n* = 528) or the Rembrandt database (*n* = 219) were compared to non‐tumour brain samples (*n* = 10 for TCGA and *n* = 28 for Rembrandt). Unpaired *t*‐tests were performed between the groups. Results are presented as means ± standard deviation (SD) of log2 TPM. Statistical significance was determined as follows: ***p < 0.001; **0.001 < *p* < 0.01; ns *p* > 0.05. (G–J) Correlations between PEA3 transcription factors levels and invasion of GSCs. (G) the levels of ETV1, ETV4, and ETV5 mRNA in GSCs (*n* = 13) analysed by RNAseq were normalised with GAPDH levels and compared using a one‐way Anova test. Results are presented as means ± standard deviation (SD) of log2 TPM. Statistical significance was determined as follows: Ns *p* > 0.05. (H–J) The correlation curves between ETV1, ETV4 or ETV5 mRNA levels in GSCs and the invasion levels of GSCs were performed with the Excel software using the RNAseq data and the invasion data previously published. The values correspond to the Pearson correlation coefficients (*R*) and their associated *p*‐values.

We compared the expression levels of ETV1, ETV4 and ETV5 mRNA between the non‐tumour brain samples and GBM samples from the TCGA and Rembrandt databases. Figure 1A,D show in both databases a significantly higher expression of ETV1 in GBM samples compared to non‐tumour brain samples. In contrast, the expression levels of ETV4 or ETV5 in GBM are significantly higher compared to non‐tumour samples only in the Rembrandt database (Figure 1E,F). We also analysed the expression of PEA3 transcription factors in low‐grade glioma (LGG) from the GBMLGG dataset of the TCGA database or the Rembrandt database. Only the expression of ETV1 was significantly overexpressed in all LGG of the two databases (Figure S1A,D). As shown in Figure S2, high expression of PEA3 transcription factors does not seem to correlate with patient survival, except for ETV5 in the TCGA database. In Figure 1G, we compared the expression levels of ETV1, ETV4 and ETV5 obtained by RNAseq performed on GSCs derived from 13 human GBM biopsy specimens. Similar levels of the three PEA3 family transcription factors are observed.

### 3.2 Involvement of ETV1 in the Invasive Capacity of GSCs

Based on a 3D invasion assay in vitro, we previously characterised the invasive capacity of the GSCs derived from the human GBM biopsy specimens used in this study [20]. From the expression data of PEA3 transcription factors and the invasion levels in the 13 GSCs, we performed correlation curves, which show a significantly higher expression of ETV1 in GSCs with a high invasive capacity (Figure 1H) while ETV4 and ETV5 expression is not significantly correlated to invasion (Figure 1I,J).

**FIGURE 2:**
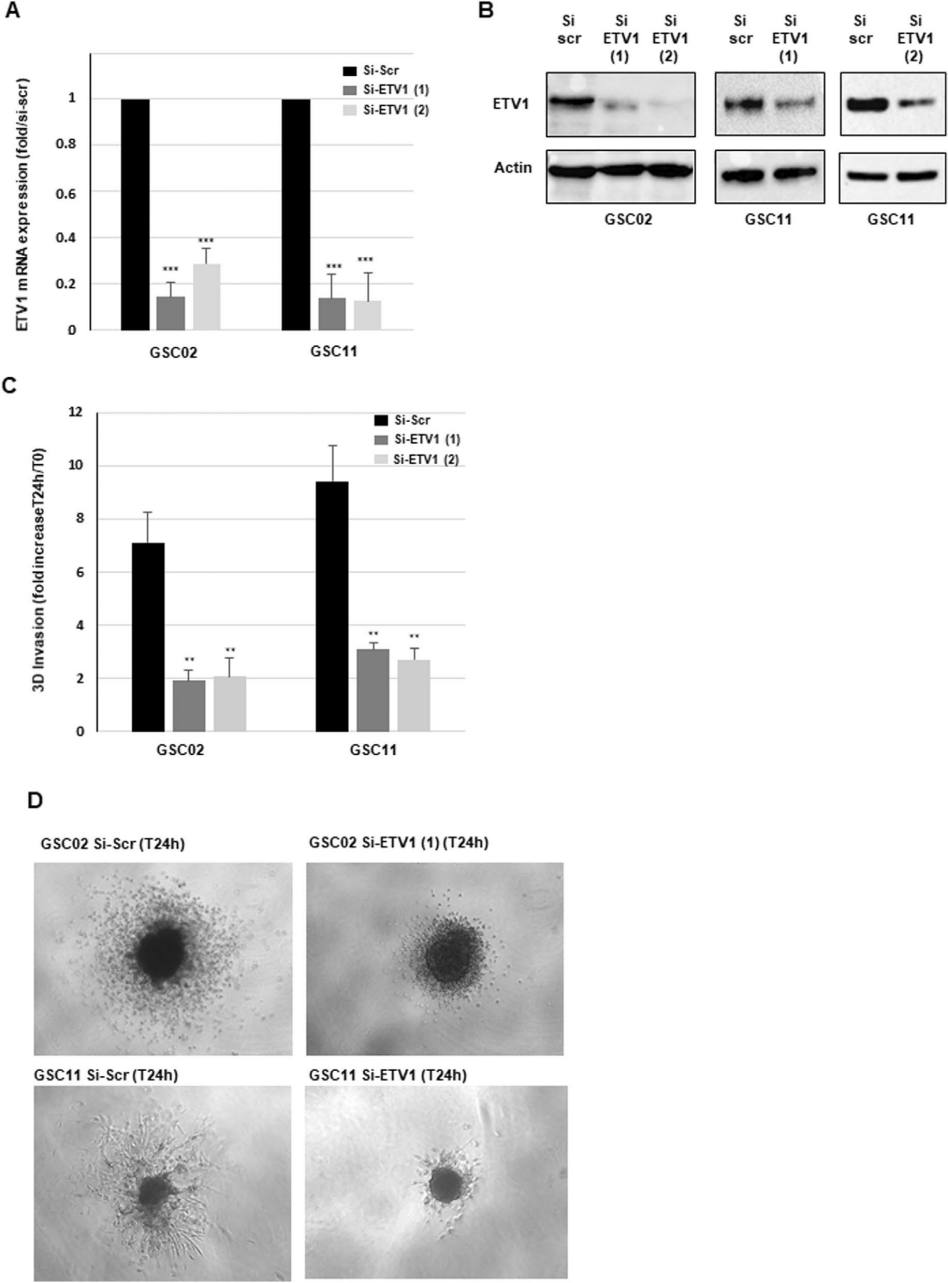
Involvement of ETV1 in the invasive capacity of GSCs. (A–C) GSCs (GSC02, GSC11) were subjected to transfection with targeted ETV1 siRNAs (si‐ETV1(1), si‐ETV1(2)) or a scramble control (si‐Scr). (A) The mRNA expression level of ETV1 was assessed using real‐time PCR. GAPDH was used as a reference gene for normalisation. (B) ETV1 protein expression was analysed by western blot analysis, using actin as a loading control. Original Western blot images are presented in the Figure S2. (C) GSCs derived from invasive GBM biopsy specimens (GSC02, GSC11) were transfected with ETV1 siRNAs (si‐ETV1(1), siETV1(2)) or a scramble control (si‐Scr) and analysed in a 3D invasion assay as described in the ‘Methods’ section. (D) Images illustrate the invasion capacity of GSC02 or GSC11 and are representative of three independent experiments. (A, C) Quantifications of three independent experiments are presented as means ± SD. A student *t*‐test was used and statistical significance was determined as follows: ***p < 0.001; **0.001 < *p* < 0.01; ns *p* > 0.05.

To our knowledge, the role of ETV1 in GBM invasion capability, more particularly in GSCs, has never been reported in the literature. To find out if ETV1 could be involved in this process, we conducted 3D invasion assays using invasive primary neurospheres derived from two different GBM biopsy specimens (GSC02, GSC11) in which ETV1 was knocked down using two specific siRNAs validated for their efficacy to inhibit ETV1 expression (Figure 2A,B). As shown in Figure 2C, ETV1‐deficient GSCs spheroids showed a significant inhibition of the invasive ability compared with spheroids transfected with a scramble control, confirming the role of this transcription factor in GSCs invasion. Figure 2D illustrates the decrease in invasion capacity of GSC02 and GSC11 transfected with an anti‐ETV1 siRNA in a 3D invasion assay.

### 3.3 Genes and Pathways Associated With ETV1 High Expression in GSCs

**FIGURE 3:**
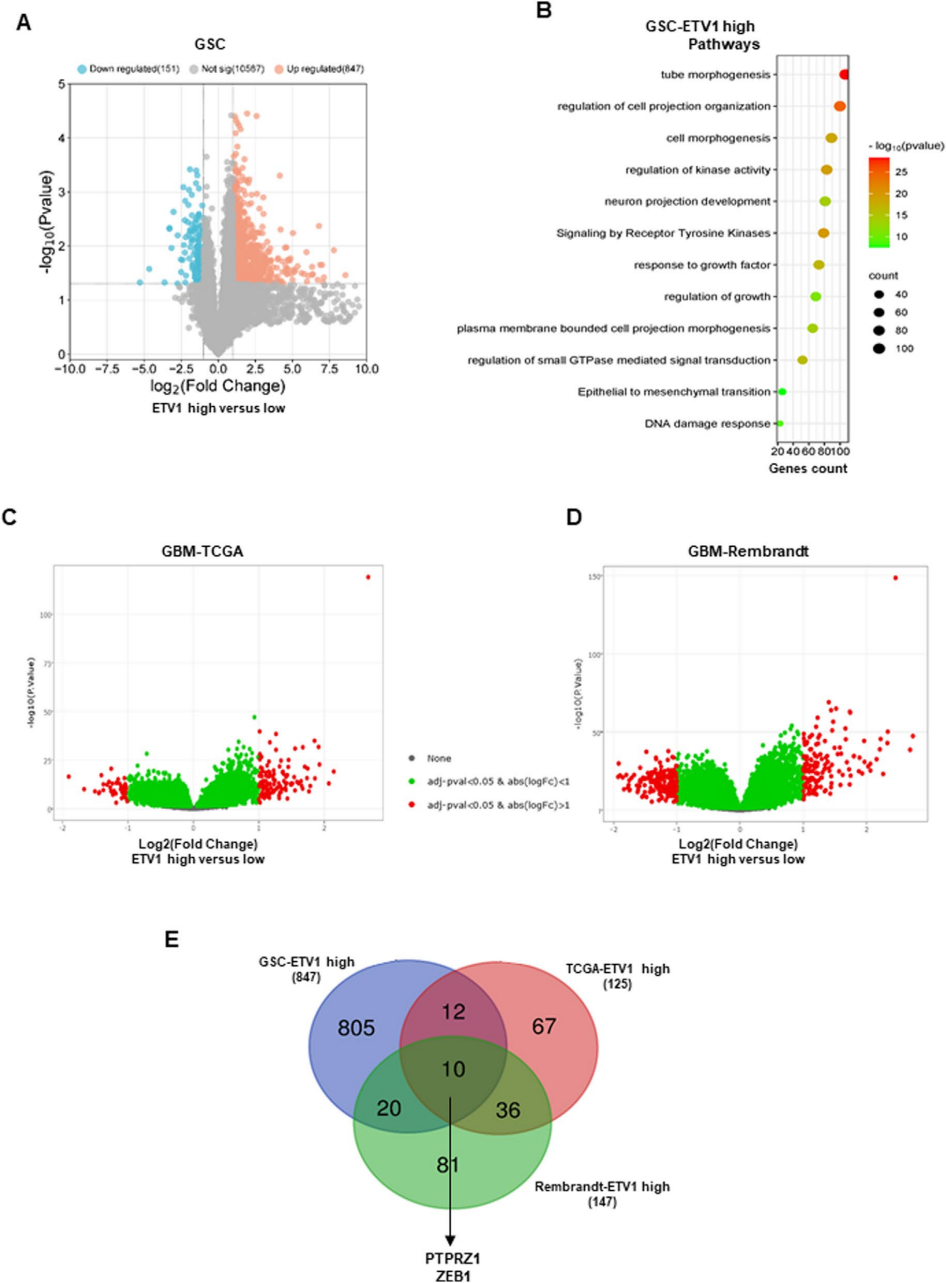
Genes and pathways associated with ETV1 high expression in GBM and GSCs. (A) A volcano plot showing the genes differentially expressed between GSCs with a strong expression of ETV1 compared to GSCs expressing weakly ETV1 was performed, using the 13 patient samples as described in ‘methods’. (B) Genes ontology analysis was performed on the genes significantly up‐regulated in the high expressing ETV1 GSCs as described in ‘Methods’. (C, D) Volcano plots generated on the Gliovis website that display the genes differentially expressed between GBM samples of the TCGA or the Rembrandt database with high ETV1 expression compared to those with low ETV1 expression. (E) A Venn diagram was performed to intersect the lists of up‐regulated genes of ETV1‐high GSCs (847 genes), ETV1‐high GBM of the TCGA (125 genes) and Rembrandt (147 genes) databases.

In order to determine the potential mechanism by which ETV1 functions in GSCs, we analysed, from the RNAseq dataset of the 13 patient samples, as described in the ‘methods’ section, the differentially expressed genes between GSCs with a strong expression of ETV1 compared to GSCs expressing weakly ETV1. The volcano plot, Figure 3A illustrates the differential changes. Using as criteria a fold change cutoff ≥ 2.0 and a *p* < 0.05, 847 up‐regulated genes were identified (red dots). The global list of the genes significantly up‐regulated is provided in supplementary Table S1. Next, pathways enrichment analysis was performed with the 847 up‐regulated genes in ETV1 high‐expressing GSCs. The results are in agreement with a potential involvement of ETV1 in the invasive capability of GSCs since several pathways related to the organisation, development and morphogenesis of cell projections as well as the epithelial‐mesenchymal transition are significantly up‐regulated (Figure 3B).

**FIGURE 4:**
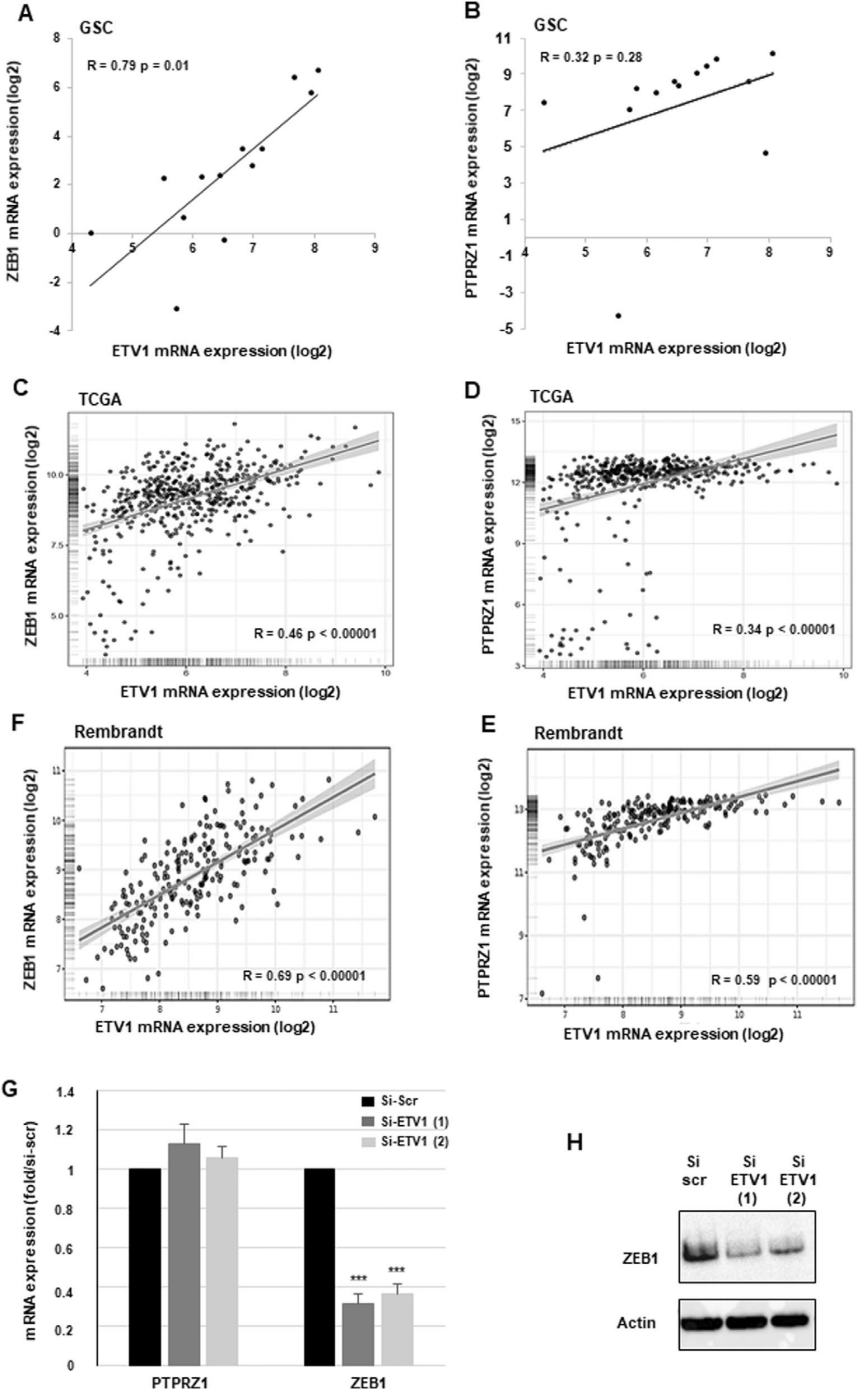
Correlations between ETV1 and ZEB1 or PTPRZ1 in GSCs and GBM of the TCGA or Rembrandt databases. (A, B) The correlation curves between ETV1 expression and ZEB1 or PTPRZZ1 in GSCs were performed with the Excel software using the RNAseq data. The values correspond to the Pearson correlation coefficients (*R*) and their associated *p*‐values. (C–E) The correlations between ETV1 expression and ZEB1 or PTPRZ1 in the GBM samples of the TCGA or Rembrandt databases were obtained by the co‐expression analysis in ‘cBioportal for cancer genomics’ (https://www.cbioportal.org/). The values correspond to the Pearson correlation coefficients (*R*) and their associated *p*‐values. (G, H) GSCs (GSC02) were subjected to transfection with targeted ETV1 siRNAs (si‐ETV1(1), si‐ETV1(2)) or a scramble control (si‐Scr). The mRNA expression levels of PTPRZ1 and ZEB1 were assessed using real‐time PCR. GAPDH was used as a reference gene for normalisation. (G) The quantifications from three independent experiments are presented as means ± SD. A student *t*‐test was used and statistical significance was determined as follows: ***p < 0.001;   ns *p* > 0.05. (H) ZEB1 protein expression was analysed by western blot analysis, using actin as a loading control. Original Western blot images are presented in the Figure S2.

On the Gliovis website, we also analysed from GBM of the TCGA and Rembrandt databases, the genes differentially expressed between GBM with a high or low ETV1‐expression (Figure 3C,D). Using the same criteria, a fold change cutoff ≥ 2.0 and a *p* < 0.05, 125 up‐regulated genes were identified in the TCGA database and 147 in the Rembrandt database (the lists are provided in Tables S2 and S3). A Venn diagram was performed to intersect the lists of up‐regulated genes in GSC and in GBM of the TCGA and Rembrandt databases (Figure 3E). Interestingly, among the 10 common genes, several genes that we and other laboratories have previously shown to be involved in the invasion of GSCs were significantly up‐regulated in the three lists [20, 23]. These genes include, in particular, ZEB1 and PTPRZ1 (Figure 3E). As shown in Figure 4A,C,F, a significant positive correlation was observed between the expression of ETV1 and ZEB1 in the 13 GSC lines as well as in the TCGA and Rembrandt databases. In contrast, while we observe a correlation of ETV1 and PTPRZ1 in the GBM databases (Figure 4D,E), no significant correlation could be demonstrated between these two genes in GSCs (Figure 4B).

Based on the correlations observed between ETV1 and ZEB1 in GSCs, we hypothesised that ETV1 might modulate the expression of this gene in GSCs. The role of ETV1 in this regulation has never been reported. In GSCs transfected with two different ETV1 siRNAs, we observed, Figure 4G,H, a decrease in ZEB1 mRNA and protein expression, which confirms the correlation between the two genes. In contrast, blocking ETV1 expression did not affect PTPRZ1 expression in GSCs.

### 3.4 Involvement of ETV1 in Radioresistance of GSCs

Since ZEB1 is also involved in radioresistance in different cancers [24], we analysed the role of ETV1 in this process.

**FIGURE 5:**
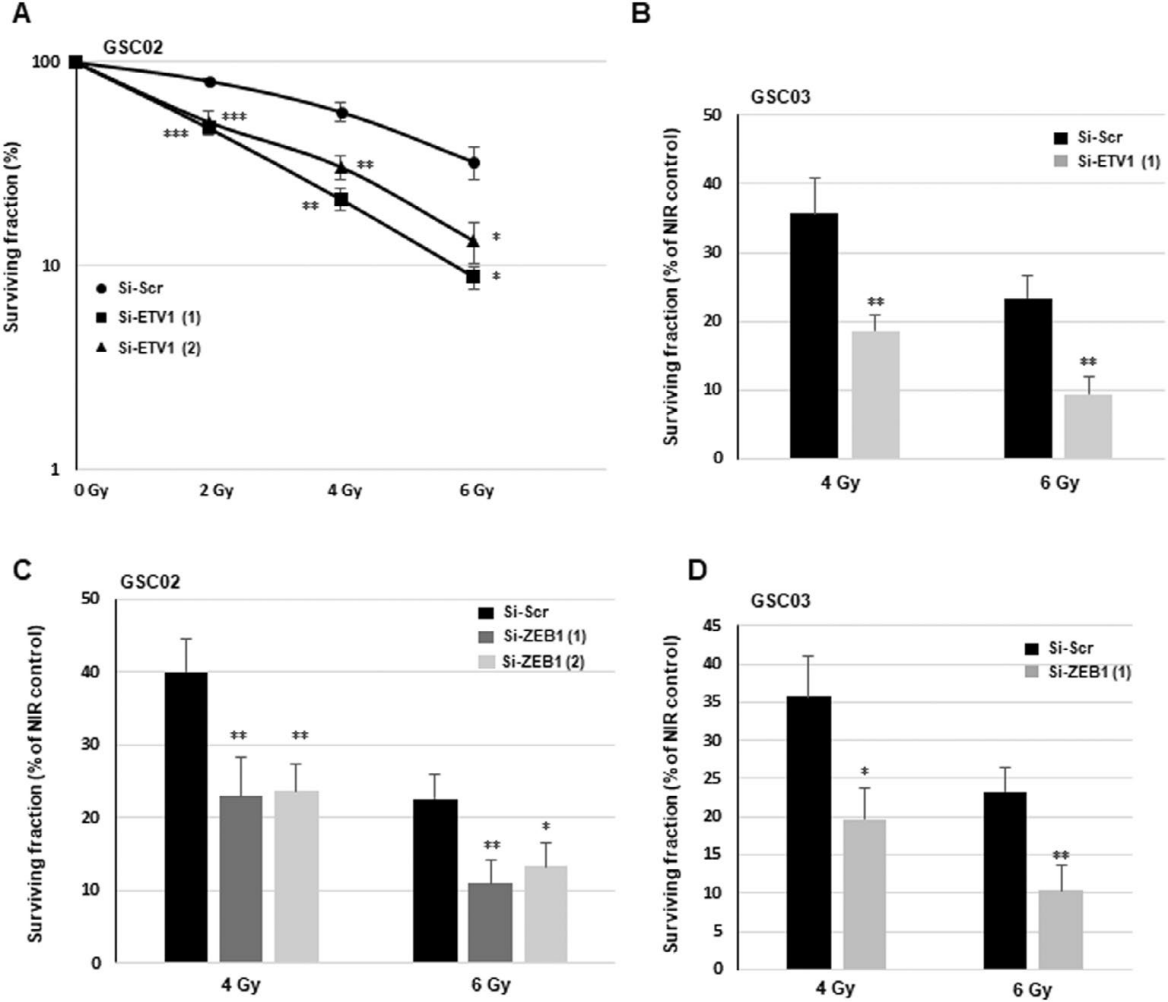
Involvement of ETV1 and ZEB1 in radioresistance of GSCs. (A–D) GSCs (GSC02 or GS03) were transfected with specific siRNAs (si‐ETV1(1), si‐ETV1(2), si‐ZEB1(1), si‐ZEB1(2)) or a scramble control (si‐Scr) and subjected to 3D survival assays with varying doses of IR (0–6 Gy) as described in the methods section. (A–D) The results, based on quantifications from 3 independent experiments, are shown as means ± SD. A student *t*‐test was used and statistical significance was determined as follows: ***p < 0.001; **0.001 < *p* < 0.01; ns *p* > 0.05; *0.01 < *p* < 0.05.

We used 3D survival assays with increasing doses of IR in neurospheres expressing two different ETV1 siRNAs or a scramble control to determine whether this transcription factor affects the sensitivity to IR. When compared to a scramble control, GSCs (GSC02) transfected with both ETV1 siRNAs showed a significant decrease in the survival fraction after IR, indicating that GSCs can be radiosensitized by down‐regulating ETV1 expression (Figure 5A). In Figure 5C, we confirmed that ZEB1 is also involved in the radioresistance of these GSCs. By using ZEB1 siRNAs previously validated for their efficiency [19], we showed the radiosensitization of GSC02 when ZEB1 expression is inhibited (Figure 5C). These results were confirmed in a second line, GSC03 (Figure 5B,D).

### 3.5 Involvement of ETV1 in GSCs Proliferation and Neurospheres Formation

**FIGURE 6:**
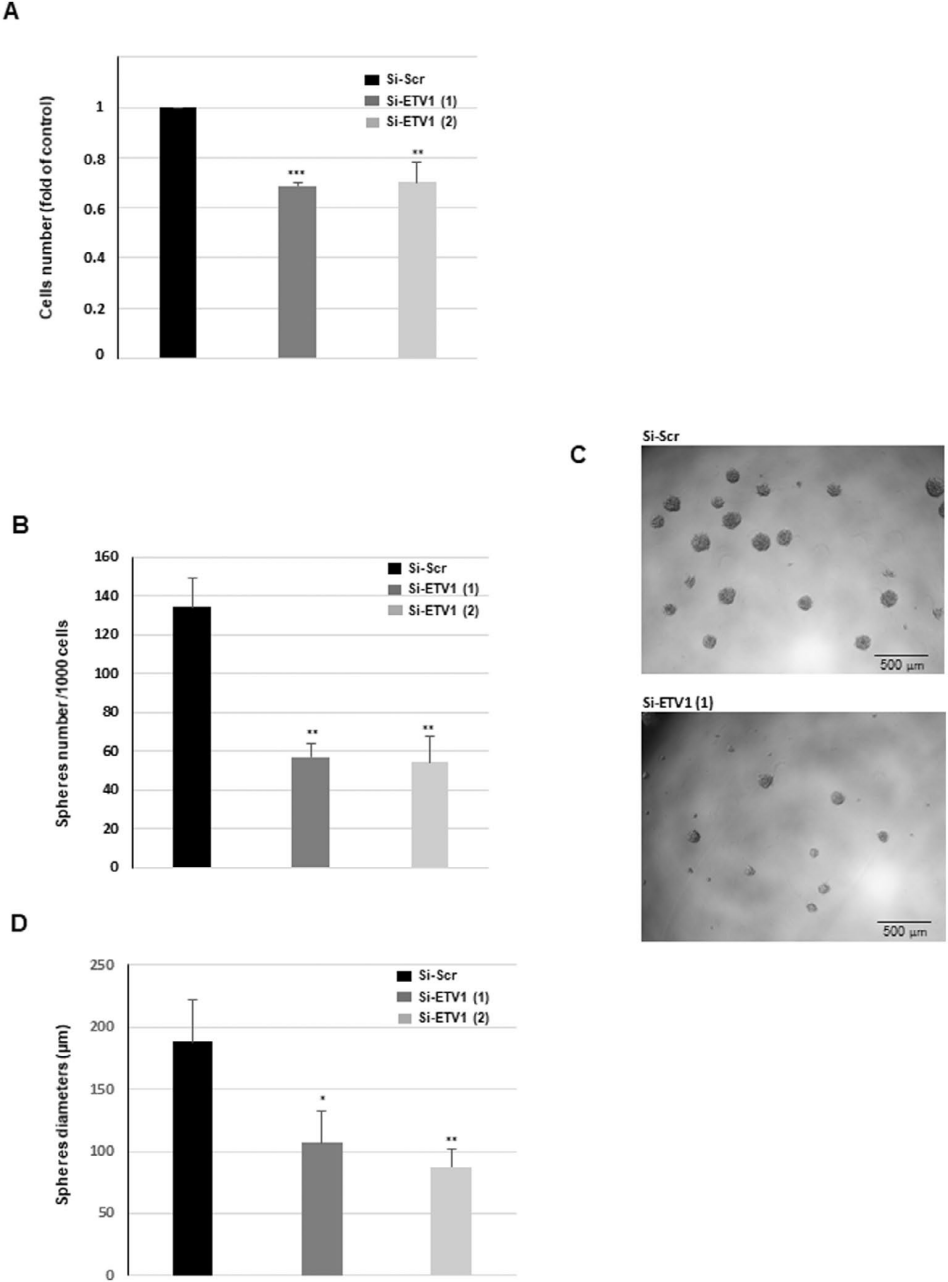
Involvement of ETV1 in GSCs proliferation and neurospheres formation. GSCs (GS02) were transfected with specific ETV1 si‐ETV1(1), si‐ETV1(2) or a scramble control (si‐Scr). (A) Cells number was determined, 48 h after transfection by cell counting using the cell counter Countess II FL. (B–D) Spheres formation was analysed as described in the ‘Methods’ section. (B) Neurospheres number was counted under the microscope. (C) Micrographs from representative fields were taken (×20). (D) The spheres diameters were measured using the ImageJ software. (A, B, D) Quantifications of three independent experiments are presented as means ± SD. A student *t*‐test was used and statistical significance was determined as follows: ***p < 0.001; **0.001 < *p* < 0.01; ns *p* > 0.05; *0.01 < *p* < 0.05.

Among the pathways significantly associated with a high expression of ETV1, we also found pathways related to proliferation and growth (Figure 3B). We therefore analysed the role of ETV1 in these mechanisms. The cell number of GSCs was significantly decreased after 48 h when the expression of ETV1 was blocked by specific siRNAs (Figure 6A). In addition, neurosphere formation was examined in GSCs transfected with ETV1 siRNAs or a scramble control. Under these conditions, we observed a significant decrease in sphere number when ETV1 was inhibited, indicating a positive role of this transcription factor in the sphere‐forming ability of GSCs (Figure 6B,C). These results were confirmed in a second line of GSCs, GSC03 (Figure S1A,B). In addition, in accordance with the inhibition of cell number observed in Figure 6A, sphere size was also decreased in GSCs expressing specific ETV1 siRNAs compared to control cells (Figure 6D).

### 3.6 Involvement of ETV4 and ETV5 in GSCs Radioresistance, Proliferation and Neurospheres Formation

**FIGURE 7:**
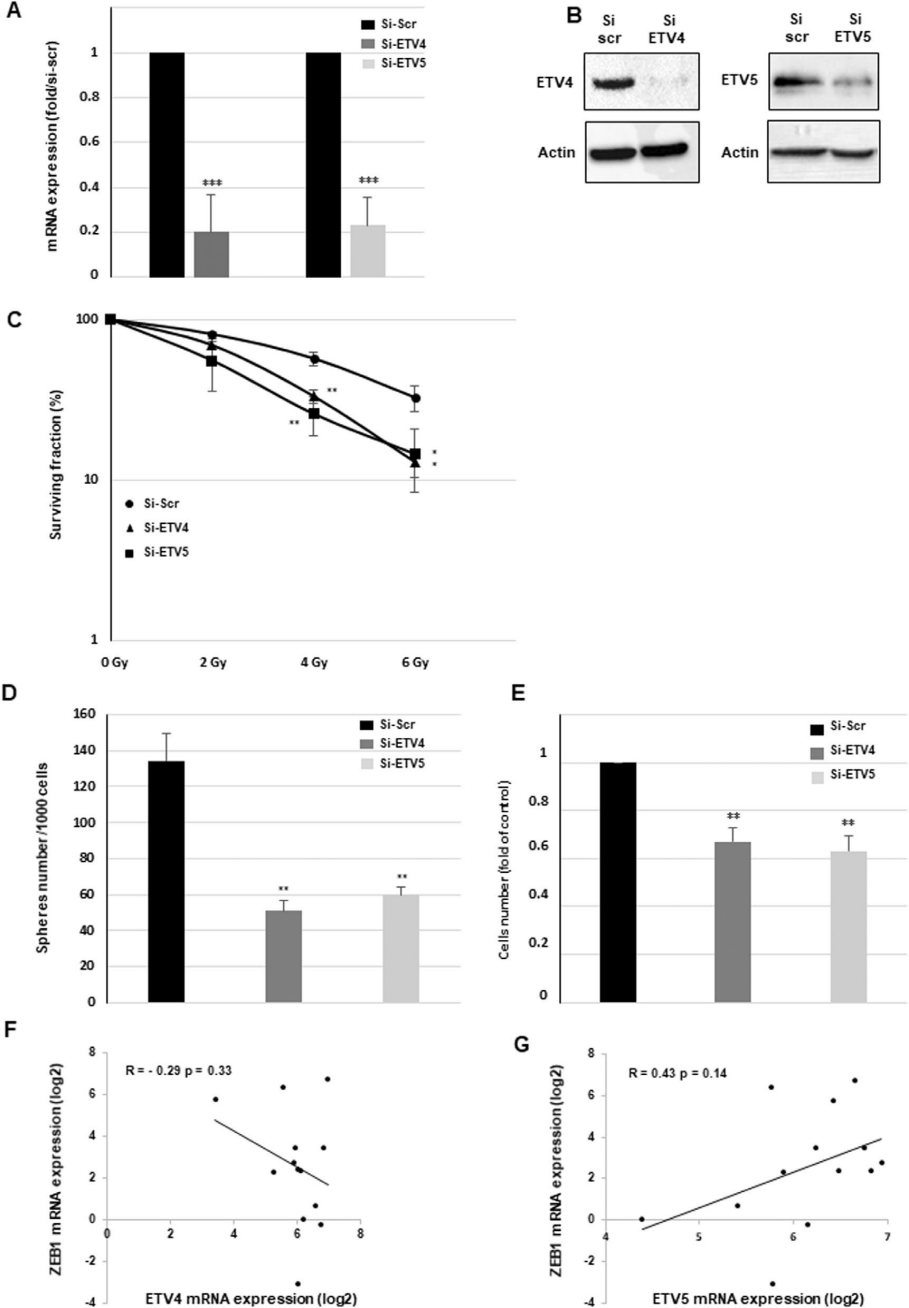
Involvement of ETV4 and ETV5 in GSCs radioresistance, proliferation and neurospheres formation. (A–E) GSCs (GSC02) were transfected with specific siRNAs (si‐ETV4(1), si‐ETV5(1)) or a scramble control (si‐Scr). (A) The mRNA expression levels of ETV4 and ETV5 were assessed using real‐time PCR. GAPDH was used as a reference gene for normalisation. (B) ETV4 and ETV5 proteins expression was analysed by western blot analysis, using actin as a loading control. Original Western blot images are presented in the Figure S2. (C) 3D survival assays were performed with varying doses of IR (0–6 Gy) as described in the methods section. (D) Spheres formation was analysed as described in the ‘Methods’ section and neurospheres number was counted under the microscope. (E) Cells number was determined, 48 h after transfection by cell counting using the cell counter Countess II FL. (A, C, D, E) The results, based on quantifications from three independent experiments, are shown as means ± SD. A student *t*‐test was used and statistical significance was determined as follows: ***p < 0.001; **0.001 < *p* < 0.01; ns *p* > 0.05. (F, G) The correlation curves between ETV4 or ETV5 expression and ZEB1 in GSCs were performed with the Excel software using the RNAseq data. The values correspond to the Pearson correlation coefficients (*R*) and their associated *p*‐values.

In contrast to ETV1, the homogeneous expression of ETV4 or ETV5 between the different GSCs did not allow us to determine two subgroups expressing high or low levels of ETV4 or ETV5 and to analyse the pathways associated with a high expression of these transcription factors. However, since average expression levels of ETV4 and ETV5 are equivalent to that of ETV1 in GSCs (Figure 1G), we also analysed the impact of blocking these two factors on GSCs radioresistance as well as the proliferation and the ability to form neurospheres. Using specific siRNAs against ETV4 or ETV5 (Figure 7A,B), we demonstrated (Figure 7C) that it is possible to radiosensitize GSCs by blocking the expression of these two transcription factors. As shown in Figure 7D, we also observed a significant inhibition of the proliferation and spheres formation by GSCs in cells transfected with ETV4 or ETV5 siRNAs. These results were confirmed in a second line, GSC03 with different ETV4 and ETV5 siRNAs (Figure S1C,D). In contrast to ETV1, ETV4 and ETV5 are not significantly correlated to ZEB1 expression in the 13 GSCs lines (Figure 7F,G) and blocking ETV4 or ETV5 expression does not reduce ZEB1 expression (data not shown) suggesting that ETV4 and ETV5 contribute to GSCs radioresistance through a different mechanism.

## 4 Discussion

GBM is a particularly aggressive brain tumour, which is also highly invasive and radioresistant. One of the causes of this aggressiveness is the existence of tumour stem cells, for which there is currently no approved targeted treatment in clinic. Therefore, understanding the mechanisms of invasion and radioresistance of these specific cells is crucial. In the present study we show in GSCs the important role of the transcription factor ETV1 in these two processes. First, we demonstrated that ETV1 contributes to the aggressiveness of GBM by favouring the invasive capability of GSCs. Indeed, we show that the expression of ETV1 in GSCs is very significantly correlated with the invasive power of the cells and that blocking the expression of ETV1 strongly reduces the invasion of GSCs. To our knowledge, this is the first report showing that ETV1 promotes the invasion of GBM cells and GSCs in particular. Furthermore, the pathways enrichment analysis carried out for GSCs expressing high levels of ETV1 demonstrates an up‐regulation of pathways linked to cell motility, supporting the role of ETV1 in the invasive potential of GSCs. Previously, the role of ETV1 in invasiveness and metastasis has been reported in digestive cancers including GIST [25], colorectal [26], gastric [27], pancreatic [28, 29] and hepatocellular carcinoma [30] as well as prostate [31, 32, 33] and breast cancers [34, 35]. In pancreatic cancer with a high ETV1 expression, the matricellular protein SPARC and the Hyaluronan Synthase 2 are two ETV1‐downstream targets responsible for its role in invasion and metastasis [28]. Classic regulators of epithelial‐mesenchymal transition (EMT), such as TWIST1, vimentin or N‐cadherin, have also been identified as ETV1 target genes involved in cell invasion induced by this transcription factor particularly in colorectal [26] and prostate cancers [31]. In addition, several Matrix Metalloproteinases (MMPs) such as MMP1 or MMP7, which regulate cancer invasiveness by modulating extracellular matrices degradation are regulated by high levels of ETV1 in prostate and colorectal cancers [32, 36]. In GSCs, blocking ETV1 did not affect the expression of these factors (data not shown). However, we demonstrated in this study that ETV1 regulates ZEB1, another master gene of EMT and metastatic processes. To our knowledge, the regulation of ZEB1 by ETV1 has never been reported. There are very few publications on the regulation of ZEB1 by the PEA3 transcription factors. Only two publications have shown a role for ETV4 in EMT via ZEB1 regulation in breast and prostate cancers [37, 38]. Although several publications have shown that PEA3 transcription factors can regulate cell invasion via the activation of major EMT genes, such as TWIST1 or ZEB1, the demonstration that they are direct target genes has not been made. In this paper, we demonstrated that high ETV1 expression in GSCs is associated with the upregulation of multiple pathways, suggesting that the regulatory potential of ETV1 might not be limited to a few target genes such as ZEB1 but might control a more global reprogramming of gene expression. In particular, we show that the high expression of ETV1 in GSCs is significantly associated with the up‐regulation of signalling by receptor tyrosine kinases, which are known to control the expression of ZEB1. In this pathway we have notably identified TGFβR, PDGFR, EGFR or FGFR which are associated with ETV1 overexpression in GSCs and which have been involved in the regulation of ZEB1 expression in previous work [39, 40, 41, 42, 43].

This study is also the first one demonstrating that PEA3 transcription factors contribute to radioresistance of cancer stem cells and in particular GSCs. Targeting ETV1, ETV4 or ETV5 in GSCs sensitised cells to radiations. The radioresistance mechanism regulated by ETV1 could involve ZEB1 whose expression is controlled by ETV1 and which is at the crossroads of EMT, metastases and resistance to therapy, including radiotherapy for many cancers. Indeed, ZEB1 is a major driver of radioresistance in breast cancer [44, 45] and plays a role in radiation resistance in several other cancers including prostate, colon and stomach cancers [46, 47, 48, 49]. In addition, we also previously demonstrated the involvement of ZEB1 in GSCs radioresistance [19]. Unlike ETV1, ETV4 and ETV5 do not control the expression of ZEB1 in GSCs, and the radioresistance mechanism regulated by these two transcription factors remains to be elucidated.

Very few bibliographic data have been published on the expression and the role of PEA3 family members in cancer stem cells. A recent publication reported that ETV1 might contribute to the aggressiveness of liver cancer stem cells by regulating LAPTM4B, a factor involved in tumour cell proliferation and metastasis [50, 51, 52]. In breast cancer, ETV4 and ETV5 are also involved in the maintenance of cancer stem cells and their self‐renewal via the regulation of sox2 or Sonic Hedgehog signalling [53, 54].

## 5 Conclusion

Altogether, the results of the present study show that the PEA3 transcription factors play an important role in the aggressiveness of GSCs, in particular ETV1, which seems to be a major driver by regulating invasion, radioresistance, proliferation and the ability to form neurospheres.

## Author Contributions

**Yvan Nicaise:** formal analysis (equal), investigation (equal), writing – review and editing (supporting). **Caroline Delmas:** formal analysis (equal), investigation (equal), writing – review and editing (supporting). **Elizabeth Cohen‐Jonathan‐Moyal:** funding acquisition (equal), supervision (equal), writing – review and editing (equal). **Catherine Seva:** conceptualization (lead), formal analysis (equal), funding acquisition (lead), investigation (equal), supervision (lead), validation (equal), writing – original draft (lead), writing – review and editing (lead).

## Ethics Statement

GBM biopsies were acquired from the Department of Neurosurgery at Toulouse University Hospital as part of a clinical trial led by Professor E. Cohen‐Jonathan‐Moyal that received approval from the Human Research Ethics Committee.

## Consent

All patients provided written informed consent.

## Conflicts of Interest

The authors declare no conflicts of interest.

## Supporting information


**Figure S1.** Expression of PEA3 transcription factors in GBM and Low grade glioma.
**Figure S2.** Kaplan–Meier survival analysis.
**Figure S3.** Complementary results on additional GSCs and controls of siRNAs efficiency.

**Table S1.** Genes significantly up‐regulated in GSCs with a strong expression of ETV1 compared to GSCs expressing weakly ETV1.

**Table S2.** Genes positively correlated to high expression of ETV1 compared to low expression obtained from TCGA‐GBM database analysis.

**Table S3.** Genes positively correlated to high expression of ETV1 compared to low expression obtained from Rembrandt database analysis.

## Data Availability

The RNAseq data presented in this study are openly available in the SRA database under the reference PRJNA1020743.
