# Supplementary material for: PEA3 Transcription Factors, Role in Invasion, Proliferation and Radioresistance of Glioblastoma Stem Cells
Source: J Cell Mol Med. 2025 Apr 24;29(8):e70533. doi: 10.1111/jcmm.70533 (PMC12022000; doi:10.1111/jcmm.70533)
Supplement: Supplementary file 1 — Figure S1. Expression of PEA3 transcription factors in GBM and Low grade glioma. Figure S2. Kaplan–Meier survival analysis. Figure S3. Complementary results on additional GSCs and controls of siRNAs efficiency. [file JCMM-29-e70533-s001.pdf]

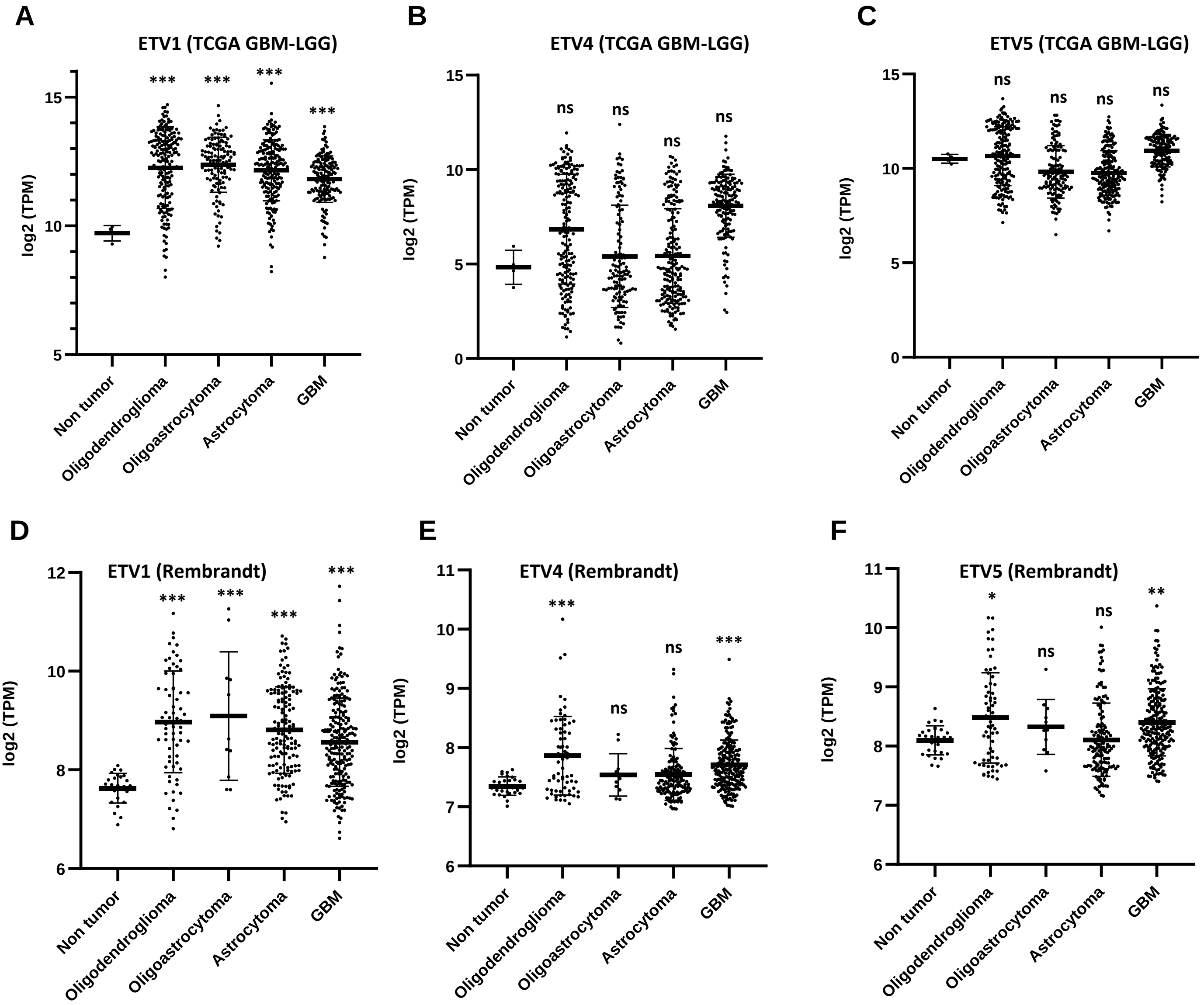

Supplementary figure S1: Expression of PEA3 transcription factors in GBM and Low grade glioma. The levels of ETV1, ETV4, and ETV5 mRNA in GBM and low grade glioma (LGG) from the GBMLGG dataset of the TCGA database (GBM n=152, LGG n=515) or the Rembrandt database (GBM n = 219, LGG n=225) were compared to non-tumor brain samples of the same database. ANOVA tests were performed between the groups. Results are presented as means ± standard deviation (SD) of log2 TPM. Statistical significance was determined as follows: *** p< 0.001; ** 0.001 < p < 0.01; ns P>0.05.

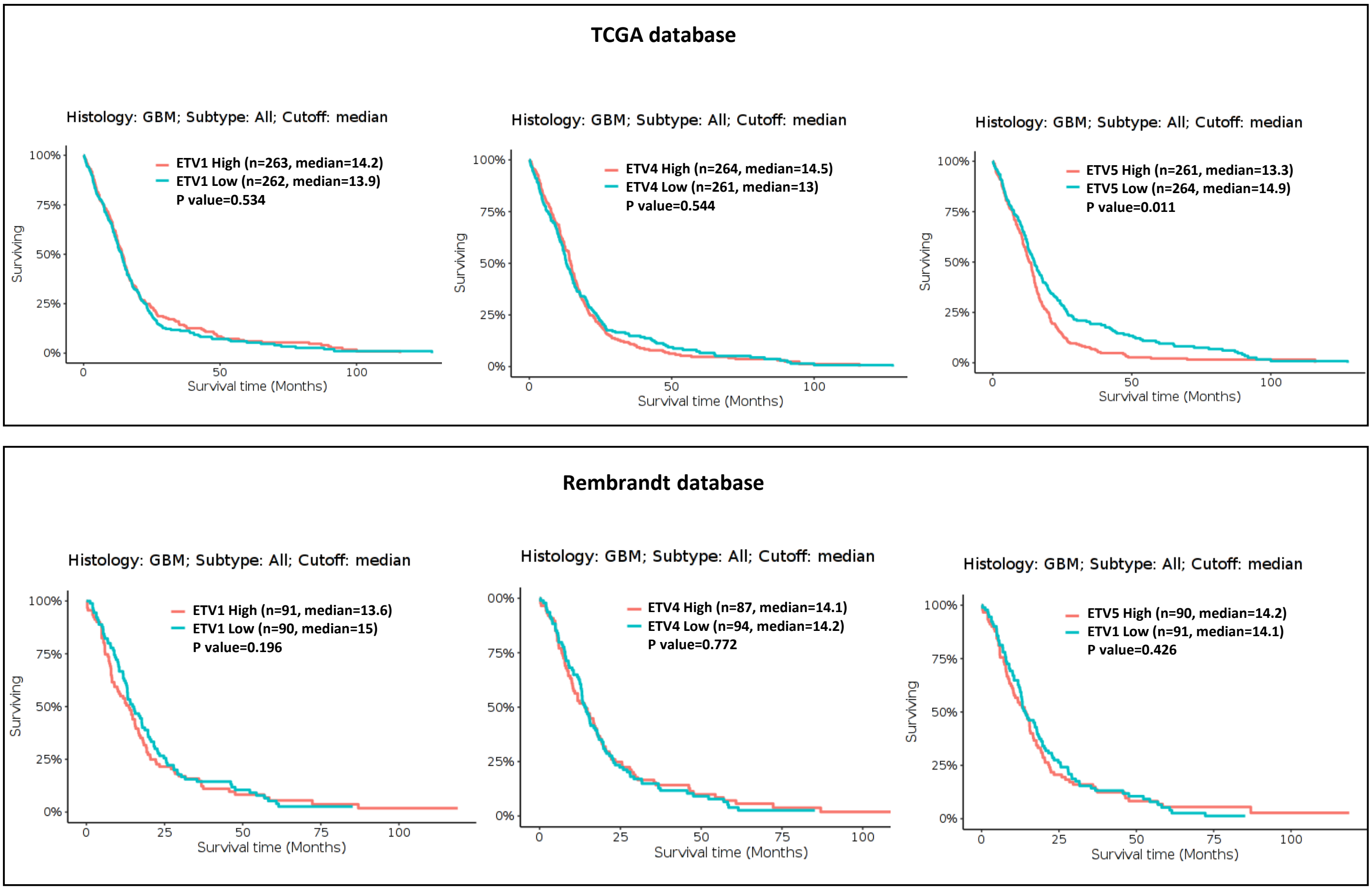

Supplementary figure S2: Kaplan-Meier survival analysis. Kaplan-Meier estimator survival analysis were performed to estimate the probability of overall survival between patients with ETV1, ETV4 or ETV5 high and low expression. P values of a Wilcoxon test are presented and considered significant when $P < 0.05$.

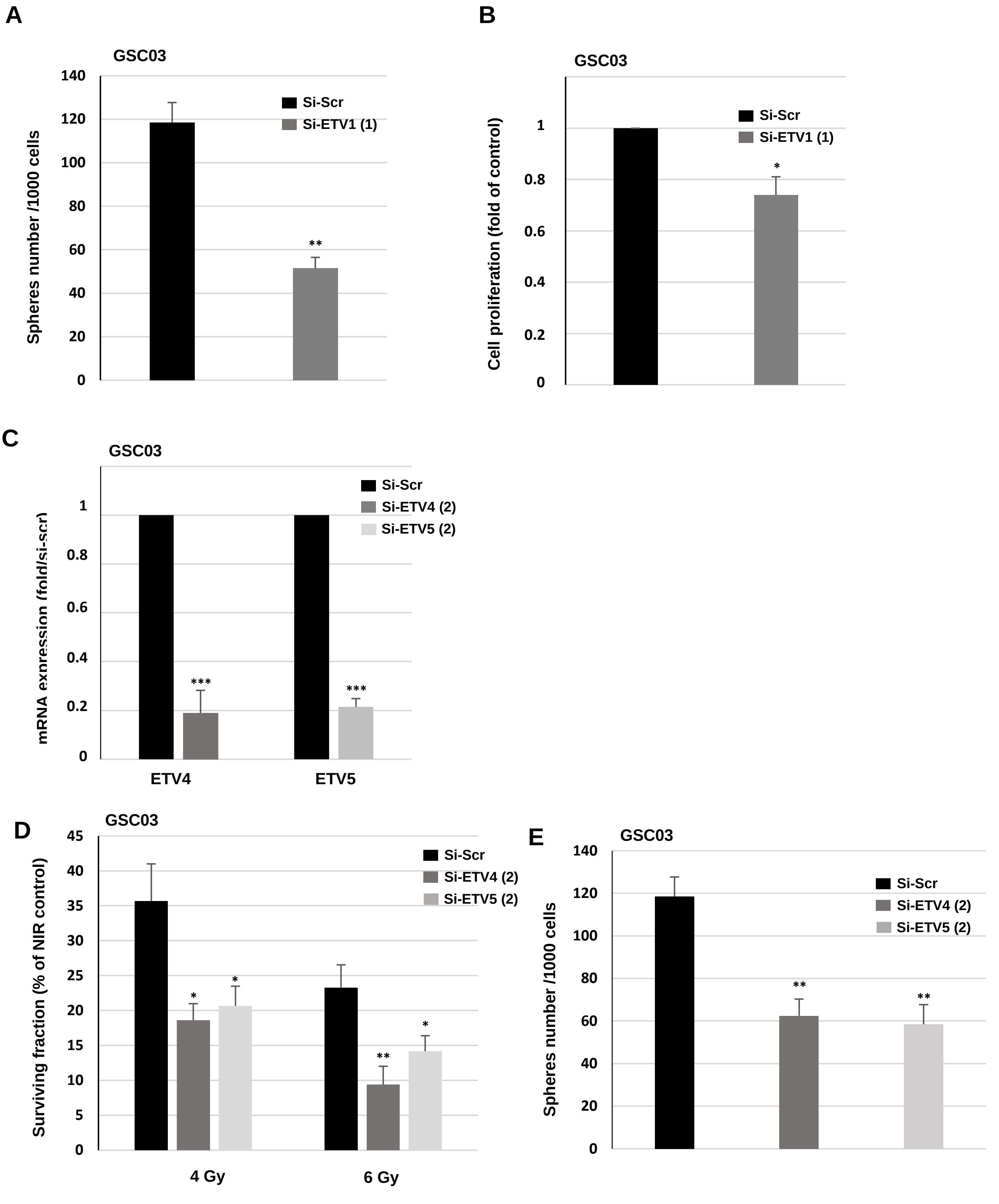

Supplementary figure S3: Complementary results on additional GSCs and controls of siRNAs efficiency. ((A-E) GSCs, GSC03 were transfected with specific siRNAs, respectively si-ETV1 (1) (A, B), si-ETV4(2) (C-E), si-ETV5(2) (C-E)), or a scramble control (si-Scr). (A-E)). (A, E) Spheres formation was analyzed as described in the "Methods" section. Neurospheres number was counted under the microscope. (B) Cells number was determined, 48h after transfection by cell counting using the cell counter Countess II FL. (C) The mRNA expression levels of ETV4 and ETV5 were assessed using real-time PCR. GAPDH was used as a reference gene for normalization. (D) GSCs were subjected to 3D survival assays with varying doses of IR (4 or 6 Gy) following the protocol described in the methods section. Quantifications of 3 independent experiments are presented as means ± SD. ***P<0.001; **0.001<P<0.01; *0.01<P<0.05.
