# Supplementary material for: PEA3 Transcription Factors, Role in Invasion, Proliferation and Radioresistance of Glioblastoma Stem Cells
Source: J Cell Mol Med. 2025 Apr 24;29(8):e70533. doi: 10.1111/jcmm.70533 (PMC12022000; doi:10.1111/jcmm.70533)
Supplement: Supplementary file 2 — Table S1. Genes significantly up‐regulated in GSCs with a strong expression of ETV1 compared to GSCs expressing weakly ETV1. [file JCMM-29-e70533-s004.pdf]

Supplementary table 1:
Genes significantly up-regulated in GSCs with a strong expression of ETV1 compared to GSCs expressing weakly ETV1 (fold change cutoff 2.0 and a p-value <0.05).

geneID	log2 FC (ETV1 high/low)	pvalue
NOVA1	1,945977542	3,53E-05
DSEL	2,58018536	3,94E-05
DBN1	1,105793336	3,98E-05
UBA6	1,186949317	4,61E-05
ZNF568	1,291550409	5,29E-05
G3BP2	1,394949495	5,89E-05
USP38	1,506093245	6,90E-05
PPM1B	1,163671142	8,15E-05
GOSR1	1,280666694	0,0001443
RALA	1,183516097	0,00019698
COPS8	1,048482432	0,00021804
PDLIM3	1,768810214	0,00024746
SP3	1,119439078	0,00030464
ETV1	1,635314113	0,00035773
CAB39	1,188850772	0,00037089
CCNI	1,154362564	0,00039953
WDFY3	1,356972807	0,00040513
SNX29	1,178545757	0,00042288
AREL1	1,310299599	0,00043706
ANKRD50	1,361889571	0,00044077
PDGFRA	4,150082147	0,0004962
PDE4B	1,603225647	0,00051892
STAM2	1,184476326	0,00056224
BMPR2	2,094190766	0,00058341
MBNL3	1,790486856	0,00059797
APBB2	1,937890013	0,00061179
NFIB	1,491690347	0,00063059
SLC30A1	1,134202161	0,00063524
SLAIN2	1,133934806	0,00066262
DCTN4	1,115234231	0,00066561
TLE3	2,025431449	0,00067897
ENAH	1,266905436	0,0006874
CLDN12	1,131432746	0,00069409
DDAH1	1,110374198	0,00070764
PLEKHA3	1,247059174	0,00075274
MBD5	1,165248514	0,00076143
KIAA1191	1,074866495	0,00077452
JADE1	1,205290733	0,00077693
CAND1	1,073335042	0,0007816
ZDHHC14	1,26489715	0,00081248
PYURF	1,456120708	0,00087383
PAK2	1,077187159	0,00091995
TMEM33	1,082143433	0,00092808
EPHB2	1,712122422	0,00093708
KRAS	1,047501406	0,00097113

ZNF416	1,281425599	0,00104422
PBX1	2,602422909	0,00108363
SIPA1L3	1,812172652	0,00112032
SOBP	2,243132018	0,00112312
ERGIC1	1,066981392	0,00120125
IDH1	1,050031314	0,00124119
SEMA5A	3,036027333	0,00126095
FBXO17	1,365406225	0,00127074
NDST1	1,211310511	0,00132699
FBXW11	1,09110857	0,00135784
ATN1	1,017428736	0,0013646
PPP1CB	1,492623493	0,00137396
ZNF480	1,005371267	0,00141384
SH3PXD2B	1,239250473	0,00148025
ARF3	1,110290926	0,00148094
TRIM23	1,155736737	0,00148574
TLK1	1,079099239	0,00150385
TRIM4	1,035083606	0,0015196
EXOC5	1,218212455	0,00153819
ITFG1	1,071875227	0,00156358
FBXL17	1,7083191	0,00158017
SNX19	1,022075803	0,00160647
PCDHB9	2,208035559	0,00161218
NAP1L5	1,319559277	0,00166016
RCHY1	1,138185568	0,00166909
ZNF134	1,250506536	0,00169304
GALM	2,035587893	0,00170311
RFX3	1,475238261	0,00172273
ZNF432	1,051082636	0,00174171
ZNF827	1,548436625	0,00174868
PCDHGC3	1,497633874	0,00178636
CHP1	1,012985002	0,0019322
DNAAF2	1,071303004	0,00196665
FKBP11	2,773948114	0,00203649
AMOTL1	1,217556124	0,00204136
CDC42SE2	1,419168983	0,00206425
CCNYL1	1,386522292	0,00213235
FRYL	1,220828037	0,00217055
TMX1	1,39821094	0,00219009
PCNXL4	1,0698037	0,00224889
ZNF304	1,039107039	0,00226163
TRPM3	1,368942914	0,00226673
KLF3	1,695811436	0,00230772
TGFB1	1,711583896	0,00230862
DENND4C	1,085730981	0,00236845
SNRPN	1,484796547	0,00238756
HS6ST1	1,087068674	0,00240211
SH3BP4	1,764150335	0,00248129
NGRN	1,12908806	0,00252391
WDR1	1,190037686	0,00254533

KALRN	3,049240231	0,00255258
LMO4	1,044897612	0,00255728
TBL1X	1,17734899	0,0026231
MEIS2	1,330585898	0,00264048
SOCS4	1,302967472	0,00272517
KPNA1	1,044325855	0,00272535
GNA12	1,381285352	0,00274993
FMNL2	1,185288974	0,0028466
PANK1	1,010377763	0,00289411
FKBP14	1,197599003	0,00291216
TMEM55A	1,111004921	0,00292157
SEC23A	1,47021464	0,00306308
HSPA13	1,155356275	0,00307202
BTBD7	1,272250063	0,00307225
CORO1C	1,134231561	0,00308069
PIK3CA	1,003486176	0,00309981
TMEM65	1,413929998	0,00310558
ZNF574	1,011896353	0,00312647
CERKL	2,013785241	0,00315955
MARCKS	1,389082608	0,00316203
PPP2CB	1,032863951	0,003267
SUCO	1,918698189	0,00327448
AP5M1	1,119350005	0,0033411
TMEM263	1,308291972	0,00334457
MAPK6	1,249196881	0,00334608
KDELR2	1,227171089	0,00336097
PSEN1	1,159048553	0,00336675
CHIC2	1,086147579	0,00337756
FAM3C2	1,267136111	0,00339732
DLGAP4	1,030811881	0,00339775
CHPF	1,21151019	0,00342393
BZW1	1,101082313	0,00349999
KPNA4	1,123176742	0,00351587
TIPARP	1,772946201	0,0035511
GSK3B	1,240834688	0,00359226
PJA2	1,461914401	0,0036408
POR	1,295084885	0,00364771
ABHD5	1,010350188	0,00365708
PRRC1	1,006371411	0,00366103
MYO1B	1,776818295	0,00367629
ZNF71	1,157055581	0,00374821
SHOC2	1,086978926	0,00376185
LIMA1	1,761925426	0,00377933
ATP2A2	1,473703327	0,00380991
TRAK1	1,057055405	0,00381352
SCAMP1	1,226319299	0,00388867
SCRN1	2,201284675	0,00397564
AGFG2	1,234459795	0,00400792
KIFAP3	1,075857443	0,00404357
HERPUD2	1,112021763	0,00408735

TMEM30A	1,135021193	0,00410017
ZNF184	1,047981087	0,00415797
CLIC2	2,113643684	0,00415855
SGCB	1,842219868	0,00417941
MOB1B	1,255269934	0,00418654
LDB2	6,78627953	0,00427453
BBX	1,161765295	0,00429531
PPP2R2B	1,80628295	0,00438849
SCYL2	1,061623095	0,00438953
YKT6	1,504134676	0,00439324
PCDHB10	2,972575055	0,00441907
ATP10D	2,282996306	0,00444231
FAM3C	1,32418171	0,00444666
CAB39L	1,389309586	0,00450351
AP1AR	1,306793427	0,00450981
ZNRF2P2	1,332240262	0,00451225
STRN3	1,260811059	0,00463529
ADCY9	1,218711328	0,00471587
DLG1	1,567686928	0,00475029
TTC7B	1,036739246	0,00478758
CEBPG	1,036279897	0,00486709
SEC61A1	1,284818593	0,00487986
POU6F1	1,200679738	0,00491402
LGR4	1,68901307	0,00496009
CDK14	4,204894717	0,00497863
NUDT4P1	1,711446634	0,00500924
NUDT4P2	1,711446634	0,00500924
GNB2	1,061584553	0,00505273
PCDHGC4	1,911033568	0,00506356
DUSP16	1,153287861	0,00512396
PIAS3	1,004253923	0,00515258
PTPRG	2,324208884	0,00531558
SERINC1	1,253398742	0,0053285
CASK	1,071984315	0,00535499
GPR161	1,267001749	0,00536353
TSC22D2	1,238052958	0,00541299
CSNK1A1	1,119948287	0,00541761
SH3RF3	1,608901266	0,00547099
FIGN	1,537989837	0,0055902
TMEM255A	2,556332602	0,0055997
FERMT2	2,279884147	0,00560872
SPRY4	1,533133106	0,00562588
RUNX1	1,680937486	0,00564839
GAL3ST4	2,69911742	0,0056715
PPP3CA	2,762462481	0,0056843
NFIX	1,060093473	0,0057571
NOTCH2NL	1,29082712	0,00576236
TMSB4XP6	5,030589252	0,00576736
NUDCD3	1,235060131	0,00578139
SALL1	2,96653022	0,00580161

UBE4A	1,269343932	0,00588036
FBXL5	1,513028464	0,00591778
DENND5B	1	0,00593139
MTPN	1,050791523	0,00595527
FAM174A	1,333260972	0,00601009
FBXO33	1,36029034	0,00602841
PURB	1,134506165	0,00605649
PPP2R3C	1,073061312	0,00606931
UBE2Q2	1,23382105	0,00610575
ZHX2	2,142120743	0,00620413
STAG2	1,494225495	0,00621578
TMED7	1,336057894	0,00641868
LIMK1	1,832076209	0,00652565
FEM1C	1,226403732	0,00654057
PTPN21	1,214466341	0,00654222
HIST2H2BE	1,769711034	0,00654832
IL6ST	1,177163684	0,00660146
TMEM8B	1,263034406	0,00660295
KIAA1715	1,271325401	0,00662316
SEMA6A	1,345414378	0,00665581
PPP2R5E	1,623750813	0,00668971
ANKIB1	1,126999772	0,00678504
ARHGEF2	2,300382598	0,00679867
MAP2K1	1,345205993	0,0068023
MAP1B	2,063774358	0,00680647
PPARD	1,184262166	0,00681384
BPGM	1,281848716	0,006859
RP11-323N12	1,599675864	0,00686753
N4BP2	1,459973648	0,00688252
ADAMTS3	2,058250026	0,00694818
CPEB4	2,429974005	0,0069513
P2RY1	1,856389572	0,00698221
ARMCX4	2,441184981	0,00699569
SEL1L	1,877178307	0,00707247
SOCS6	1,300213566	0,00713421
SLC39A9	1,148110991	0,00715643
FOXN3	1,541914425	0,00715712
SOS2	1,461717096	0,00716038
RGL1	1,330493424	0,00718311
CDK6	1,886659346	0,0071975
ADAM9	1,421758826	0,00722444
RFFL	1,061828528	0,00722773
CSNK1G1	1,036523653	0,00724327
ZNF415	1,362419978	0,0072536
MGAT2	1,733246235	0,00727828
SOS1-IT1	1,115776315	0,00732468
IRF2BPL	1,909499056	0,00740859
PGRMC2	1,041672153	0,00741999
NCOA3	1,043143841	0,0074511
GTF2IRD2	1,386531072	0,00753634

RAB2B	1,231515868	0,00755756
RAB21	1,082295131	0,00760458
CSRNP2	1,212517282	0,0076524
BCAR1	1,124124192	0,0078613
BAG5	1,320718248	0,00793112
PHF12	1,062619962	0,00802899
SPATA7	1,014362895	0,00804535
FAM69A	1,25973581	0,00813512
PTP4A2	1,098545458	0,00814346
GSKIP	1,697923393	0,00816159
ZBTB44	1,56145333	0,0081662
APOLD1	1,256039507	0,00816966
AFAP1	1,692850356	0,00826157
TSPAN11	3,304890269	0,00835799
VOPP1	1,509455413	0,00838303
PDGFC	1,655530126	0,00841111
ZNF816	1,105832912	0,00841582
CDC42BPA	1,207201328	0,00851701
FADS2	1,655181805	0,00860649
CCDC109B	1,438319682	0,0086658
ZFAND3	1,242325256	0,00872358
C3orf58	1,509760924	0,00872368
KLF6	1,237507748	0,00872847
BACE1	2,340570481	0,00874675
AFF4	1,069904099	0,00875779
PRKACB	1,435644976	0,00883109
CDV3	1,071484135	0,00890327
MCL1	1,210071736	0,00892307
ZNRF2P1	1,510091959	0,00909037
CDR2	1,06637312	0,00918459
MBOAT7	1,074270765	0,00919008
HERC4	1,073429618	0,00921028
NHSL1	2,084759237	0,00925121
AVL9	1,428471417	0,00929383
TEAD2	1,268214645	0,00948273
KANK2	1,415876219	0,00948835
ATP13A3	1,5271712	0,00950803
CORO2B	1,238966341	0,00957115
ADAM12	2,695888058	0,00957424
PPP1R3F	1,00059297	0,00960326
ITM2B	1,327713136	0,00968576
TNFRSF21	2,250890971	0,00974086
RAB31	1,353337325	0,00975868
PTPRN2	1,168775818	0,0097632
PHF1	1,324834837	0,00982555
AGPAT4	2,579642169	0,00985502
RAB33A	2,556515154	0,0098803
ZBTB4	1,060368666	0,00991142
RGS17	2,179726735	0,00996611
DNMBP	2,28553262	0,00998395

PDP1	3,023146703	0,00998459
MEF2C	1,732557579	0,01003838
TM9SF1	1,481991116	0,01017199
UBE2J1	1,284086813	0,01021453
SLC12A6	1,128223929	0,0102273
TAP1	1,352388637	0,01026386
PARP9	1,327785658	0,01027
HEG1	2,76156244	0,01027123
LRRFIP2	1,143422365	0,01033601
MAGI2	2,295114099	0,01050283
TPST1	1,875521597	0,01050414
DENND2A	4,593128557	0,01058294
SNAPC3	1,498476558	0,01058847
SLC16A2	1,947291157	0,01059867
SPRED1	1,150717762	0,01068809
ZEB1	4,503211931	0,01076284
TCF4	1,489286287	0,01094364
IGF2BP3	1,624712249	0,01094748
PANK3	1,366697575	0,01096971
METTL7A	3,504647029	0,0110018
MGAT1	1,081457523	0,01106587
CC2D2A	1,486435529	0,01106606
ZNF615	1,014565842	0,01110301
RRAGA	1,090836283	0,01112503
CD276	1,212341399	0,01113281
NDFIP1	1,423643623	0,01119648
TINF2	1,24329204	0,01123867
SLC36A4	1,349200023	0,01126232
CCM2	1,203645565	0,01145128
DYRK3	1,293752991	0,01149965
CPD	1,084866513	0,0115525
SOAT1	1,092858381	0,01159002
KPNA5	1,279939444	0,01162361
EDEM3	1,320596741	0,01167762
TAOK1	1,064025656	0,01169746
MTF1	1,016663122	0,01169853
LRIF1	1,154167225	0,01170804
CAMK2D	2,842423421	0,01171626
MSMO1	1,072558914	0,01190428
POSTN	7,792255002	0,01193567
NR3C1	2,138479624	0,01197607
DNAL1	1,132783688	0,01201629
SHC3	2,152078037	0,01203093
PCDHB8	2,344771123	0,01205907
TMEM185B	1,420710465	0,01212316
SCD5	1,241213244	0,01214385
KLHL5	1,358552719	0,01216395
STYX	1,25042493	0,01225757
XYLT1	2,557520616	0,01228348
F2R	3,324540009	0,01247201

STAMBPL1	2,558815569	0,01256982
HBEGF	1,471712379	0,01264818
ZNF239	1,743339	0,01268623
TULP4	1,112823817	0,01269841
SMIM14	2,120094587	0,01270615
RGAG4	1,81924902	0,0127514
GALNT4	3,422826301	0,01291889
PFN2	1,177980608	0,01292168
SPOCK1	5,947347691	0,01299116
SATB2	2,234592152	0,01302256
CHST14	1,387996757	0,0130767
ZNF175	1,005744936	0,01308441
SOS1	1,087327052	0,01312182
KLHDC2	1,107087058	0,01312818
SIPA1L1	2,523499779	0,0131492
HOXA3	1,765185878	0,01318174
SLC2A10	2,56496376	0,01321823
PNMA1	2,060320946	0,01322038
JUN	2,664156406	0,01324554
PRR4	1,195067427	0,01335106
DFNA5	2,29769693	0,01360212
PAPOLA	1,086477117	0,01364587
SIRT1	1,033671732	0,01365957
SLC4A7	1,201691541	0,01372212
ADGRA3	1,50127856	0,01388219
APH1B	1,364471201	0,01394629
PDGFRB	1,86085946	0,01395287
CYP51A1	1,157032505	0,01400136
GNS	1,127749844	0,01404212
GADD45B	2,570297114	0,01412577
TSC22D1	2,980059006	0,01417912
CMTM6	1,042419303	0,01428481
RAB43	1,609508799	0,0143521
ITGA4	1,924823532	0,01438354
FCHSD2	2,283293245	0,01441147
MYL6	1,185808861	0,01442435
C14orf37	1,971534688	0,01450713
DHRS7	1,43724576	0,01452653
CD164	1,445841278	0,0145539
OSBPL6	1,181327238	0,01457363
VPS37D	1,257667896	0,01458543
PPP1R2	1,506246828	0,01460237
NOTCH2	1,558743819	0,01467112
CYP2U1	1,667719725	0,01478089
UBE2H	1,821429655	0,01494272
ZNF835	1,458268826	0,01495482
CCDC91	2,938887349	0,01502733
BCAS4	1,11149055	0,01507573
GLRX	1,91003687	0,01509128
DAAM1	2,166301815	0,01509147

DLG5	1,286356494	0,01520845
ZNF177	1,343751903	0,01528642
VAMP4	1,059538808	0,01537941
SEC24D	1,206346904	0,01542315
ANXA5	1,543771787	0,01549381
RP11-717F1-2	1,332268231	0,01551753
NFATC4	3,143921426	0,01556176
EPS8	1,030335545	0,01556617
NLGN2	1,070146046	0,01562281
LIMCH1	1,197031329	0,01567543
PHKB	1,155754165	0,01570384
PKNOX2	1,712260778	0,01573588
LRP10	2,587646416	0,01574926
KIF5C	2,199809281	0,0157543
DOCK4	3,100562209	0,0157739
DTX3L	1,732946672	0,01585232
PLIN3	1,340635221	0,01586342
CTSB	2,058356702	0,01596382
TGFB1I1	1,15182613	0,01600454
ADGRL3	1,995027067	0,01602844
SGPP1	1,718521494	0,01603461
RFX2	1,357509762	0,01604696
AFAP1L1	1,436580549	0,01609334
GRAMD1B	2,349242548	0,0160972
PGAP1	1,293965354	0,01613143
RAP2C	1,349466324	0,01623575
FAM20C	2,266769528	0,01625463
SPTSSA	1,366233697	0,01627392
PEA15	1,157082985	0,01655456
UBE2E3	1,107484835	0,01657861
TRAPPC6B	1,313387861	0,01667016
ZNF503	2,285487912	0,01672237
ATP6V1D	1,089412562	0,0167286
EMP3	2,414643406	0,01673652
PLIN2	4,041796418	0,01675984
ANK2	1,82122141	0,01683226
GNAI1	1,417615624	0,01686754
HTRA1	2,996036285	0,01690622
SGMS1	1,138011783	0,01693875
ATP2B4	2,29904883	0,01696796
EMILIN1	4,865832267	0,01703028
NPAS2	2,875567827	0,01715341
C6orf120	1,027451538	0,01723514
ACBD7	1,546658613	0,01733344
ANGPTL2	1,041896621	0,01733954
TRPM4	1,562920169	0,01737831
EPHA2	3,246705432	0,01738457
BACH1	1,122757544	0,01738697
CGRRF1	1,183503885	0,0174806
WFS1	1,065087355	0,01753898

DDAH2	1,31167079	0,01756183
TACC2	3,160531866	0,01756362
IL17RD	2,589340941	0,01759271
GFPT1	1,069809194	0,01763415
B2M	1,233782644	0,0176837
RNASE4	2,398913472	0,01782454
TENM4	3,993242634	0,01784948
ZFAND2A	1,267437434	0,01786222
SLC1A3	4,70152201	0,01793984
JADE2	2,644850064	0,01803522
C10orf10	4,712999488	0,01813694
BOC	3,563656491	0,01824239
AZIN2	1,26654889	0,01830306
AIG1	1,062088301	0,01833678
SMAD1	2,631388507	0,01843873
TSPYL1	1,131676477	0,01848994
UBASH3B	1,588650688	0,01852898
TIMP4	2,595745615	0,01854775
IDS	1,479439968	0,01855779
COG5	1,118783728	0,01858613
DCAF5	1,199114784	0,01858693
EHD2	2,992349168	0,01859531
GPM6A	4,804802083	0,01865362
USP13	1,487152281	0,01875486
ZFP1	1,069991552	0,01879696
DPY19L2P3	1,451079538	0,01884098
B3GNT5	1,847057346	0,01884619
CSF1	2,605390452	0,01884921
RTN4	1,704706447	0,01892637
PI4K2B	1,107602955	0,01894795
DHRS4	3,533963552	0,01900376
PSMB9	1,09184178	0,01909637
CSGALNACT1	4,313207941	0,01916273
C16orf72	1,047706057	0,0191769
BCAP29	1,41063208	0,01918313
SMAGP	3,49660855	0,01923076
KCTD12	1,847886554	0,01928541
RND2	2,54182178	0,01930192
PLOD2	1,289064227	0,01941018
RAB23	1,239405924	0,01953586
ARHGEF3	3,309988482	0,01964298
BTN2A1	1,129271881	0,01964933
RCOR1	1,191200448	0,01973647
PCDHGA2	3,267003308	0,01976299
MPP6	1,456899012	0,01981032
KMT2E	1,22023097	0,01993735
TMBIM6	1,087945498	0,02000361
SALL2	2,389697912	0,0200192
TMOD3	1,493188989	0,02005888
CACNA2D1	3,165423609	0,02024978

ZBTB38	1,266766273	0,02027097
NUDT4	1,64297508	0,02047366
MAP4K3	1,029735399	0,02051709
EPHA4	1,072851686	0,02061124
HIST1H2BD	1,390058332	0,02063731
TMEM43	1,087659998	0,02070142
PSD3	2,919437893	0,02077279
RAB30	1,091779603	0,02097532
MCC	3,202218872	0,02124528
FLT3LG	1,096623311	0,02136358
TSHZ2	2,19588409	0,02137782
CD302	1,090908073	0,02145026
NADK2	1,365757418	0,02146901
SSBP2	1,737414862	0,02148808
ZBTB20	1,01485861	0,02155912
ABL1	1,211309255	0,02168389
LNPEP	1,155045438	0,02170406
PLCD3	1,469470725	0,02192428
CLIC4	1,192698978	0,02197738
TSPAN5	2,783154486	0,02204099
SPIN1	1,006551969	0,02208237
GLI3	1,054755428	0,02214656
DPYSL3	2,089529891	0,02216062
HECA	1,641832762	0,0222258
SPOCD1	6,8657209	0,02224004
ZNF703	1,201026093	0,02224954
APP	1,439115261	0,02229237
HLA-C	1,558932527	0,02236338
CPSF2	1,006763746	0,0223643
C1orf54	2,640324499	0,02243342
CCDC80	6,998233573	0,0224353
TSPAN7	1,162964005	0,02245011
SLC41A2	1,096525668	0,02245045
RASD1	4,518604434	0,02271595
PVRL2	1,421571035	0,02272015
ERAP1	1,644850969	0,02283808
APLP1	2,221131151	0,02285993
TFPI	3,085084679	0,02286771
HDAC7	1,216721699	0,02290926
RDH11	1,096215315	0,02316776
PRR5L	2,230051372	0,02330232
COLGALT2	1,668285468	0,02334124
LIN54	1,005866177	0,02337302
ZBTB47	1,006396042	0,02338152
KIAA1671	1,387524272	0,02340949
ANO6	1,083371653	0,02350081
SCG2	5,350779161	0,02362453
CBR3-AS1	1,099734881	0,02362937
NR1D2	1,055303834	0,02371432
FCGR2A	1,214264052	0,02383976

MTCH1	1,196222315	0,02385402
MAP4K5	1,111010092	0,02394963
HIVEP3	1,547737814	0,02419827
ZMAT3	1,852824483	0,02426985
CREB3L1	6,32356659	0,02427099
SDC2	3,701590969	0,02442828
FAM132B	1,041746511	0,0244851
SKIL	2,293722831	0,02453756
UBE2L6	1,258776134	0,02464951
TMEM158	2,640006516	0,02468433
CALU	1,215247808	0,0248007
SNX18	1,788993719	0,02499544
ZHX1	1,17647402	0,02505168
NREP	3,00410546	0,02512011
PKIB	3,961576695	0,02516634
PALLD	3,44096633	0,02516902
STK40	1,045361978	0,02519101
MIR99AHG	1,809088259	0,02530117
WLS	2,014313209	0,02535637
TMED7-TICAM	2,71270554	0,02546866
C6orf1	1,130302014	0,0255145
ARSJ	1,743229896	0,02560258
SPP1	3,326313546	0,02563204
ABI2	1,075175632	0,02565164
NRG2	1,770851157	0,02576025
TIMP2	1,321730282	0,02582611
ANAPC13	1,109442016	0,02585948
PTPN14	1,612512288	0,02596912
PPP1R3B	2,027685389	0,0260104
EPAS1	4,646972163	0,02602558
PLPPR2	1,132867114	0,02605981
ZNF530	1,124102417	0,02606048
LDLR	1,035244128	0,02607737
FICD	2,086156644	0,02611696
HLA-A	1,446361451	0,02612125
SLC38A2	1,604040318	0,02627625
CHST2	2,17725421	0,02628213
CPVL	1,943187645	0,02628778
EFEMP2	3,039968671	0,02632845
RAP1B	1,000732647	0,02633078
MYD88	1,616906223	0,02640383
TUBA1C	2,017246626	0,02642894
RALGAPA1	1,165279668	0,02648252
CDH2	1,045586328	0,0265153
ADAM22	1,316952212	0,02653981
TMTC2	1,056271024	0,02655933
SOX15	2,39160792	0,02660962
PRUNE2	2,90289328	0,02693904
PCDHB7	4,678681023	0,0269604
COQ10B	1,041518425	0,02697891

TOX4	1,035386607	0,02699407
C10orf11	2,897833111	0,02712447
SIK2	1,457735935	0,02730126
STARD3NL	1,084496887	0,02738181
PTK7	1,176152089	0,02742206
PCDHGB6	5,050820199	0,02745372
CSGALNACT2	1,248722268	0,02759316
CRIM1	3,058286828	0,02770181
CTSO	1,871510501	0,02772191
ARHGAP24	1,252598672	0,02789343
CAMK2N1	5,188351923	0,0281348
VCAN	1,320368163	0,02817874
IRS1	2,721600061	0,02817915
CAP2	1,90109461	0,02824453
EPB41L1	1,054272199	0,02828894
FCGR2C	1,881023898	0,02832199
SHROOM3	1,491126115	0,02835892
PCDHB5	4,51119061	0,02844021
ZNRF1	1,167237002	0,02851649
STBD1	1,996874866	0,02857221
CCSER2	1,116537005	0,02857382
MEST	2,583300888	0,02864347
CPT1C	3,557765357	0,02867752
ANKRD6	1,246081273	0,02875256
FBLIM1	2,611936271	0,02885489
PTPRF	1,308293175	0,02900711
NGEF	2,863550424	0,0290828
IFRD1	1,0475201	0,0291429
XXYLT1	1,159387466	0,02921241
FNBP1	1,190120439	0,02924235
RHOBTB3	1,129573731	0,02926079
ZNF461	1,033571635	0,02929001
SYBU	2,881599199	0,02940575
WNK1	1,165396656	0,0294319
C7orf60	1,014684241	0,02944238
JDP2	1,496991579	0,02945725
TUG1_1	2,823435214	0,02949751
E2F7	1,377213758	0,02962347
S1PR3	2,377348021	0,02965869
TRAM2	1,207828522	0,02979734
ENG	3,019711951	0,02995825
CHRD	2,196203885	0,0299596
JAK2	1,72269331	0,03004794
HOXD10	4,320725348	0,03021021
RAB3IP	1,566795788	0,03026468
TMSB4X	5,108886289	0,0305291
RRM2B	1,108318784	0,03056434
FNDC4	1,819731151	0,03059459
SMPD1	1,220300685	0,03063521
NEK11	1,548152977	0,03121611

CALD1	1,043002029	0,03122849
ARF6	1,494301315	0,03125315
MAN2B2	1,568724036	0,03126166
GLB1	1,097316022	0,03126443
SPEG	3,531718748	0,03132647
SYT1	2,685382992	0,03146363
ABHD2	2,301054922	0,03146829
SLC11A2	1,338236853	0,03148115
SNAP25	5,609801871	0,03150989
SPRY2	3,052077778	0,03162584
LHFPL2	1,471699989	0,03167073
SAMD8	1,134980921	0,03171966
PUS7L	1,156768387	0,0317462
NCS1	1,137755177	0,03176297
BMP2	3,306345143	0,03179877
EEPD1	2,162228195	0,03185365
SMAD9	1,14829821	0,03193158
FLOT1	2,883669385	0,03196699
SIK3	1,925753438	0,0322221
ZNF880	1,27134032	0,0323545
DSE	1,125067603	0,03240433
ABL2	1,469885976	0,03258494
PAXIP1-AS2	1,520338139	0,03262194
S100A16	4,906169735	0,03263294
HOPX	4,655861255	0,03264252
NOTCH3	2,896722113	0,03269038
TAP2	1,017906095	0,03271907
SYT17	1,284311065	0,03278875
ARHGEF12	1,248793883	0,03286485
EFR3A	1,005804087	0,03295716
WNT5A	5,790877025	0,03300389
THSD7A	4,634431177	0,03302392
ZNF300	1,168958048	0,03311355
DDR2	3,597059718	0,03313881
NPNT	6,368480784	0,03314295
ATL1	1,927147262	0,03338562
OSMR	2,864319599	0,03349953
RHBDD2	1,57782784	0,03351352
TMEFF2	2,750857435	0,03382101
BCAT1	1,922011926	0,033835
PPTC7	1,386203736	0,03389136
LETMD1	2,789606555	0,03390506
NRP1	6,327403076	0,03399132
TRIB3	1,405354103	0,03422496
GAS6	2,674080283	0,03450859
FZD2	1,371890707	0,03451819
HRH1	8,57198012	0,03461711
PRNP	1,622173327	0,03463519
PBX3	1,178984068	0,03465633
SIRPA	1,085530859	0,03467881

ZNF22	2,36394016	0,03499455
TRERF1	1,73248926	0,03500468
TJP2	2,295580004	0,03501551
ACVR1	1,430980998	0,03515793
HLA-B	1,487266979	0,03521336
POU3F3	3,464042957	0,03521665
STT3B	1,033575991	0,03526266
LAMB3	6,069673528	0,0353979
XXbac-BPG24[illegible]	1,550334664	0,03555542
MAN2A1	1,937399092	0,03568928
HELZ2	2,043091741	0,03572345
ARL6IP5	1,53878963	0,03581286
SMURF2	1,223116913	0,03584261
DHRS2	2,3267057	0,03594269
FHL1	1,468900351	0,0359574
CRISPLD2	1,676157247	0,0359759
KCTD10	1,004896393	0,03615736
HOXA7	2,123689331	0,0362835
ZNF607	1,049704301	0,03637364
FOXD1	1,591634681	0,03640027
PEAK1	1,113420449	0,03645878
PDE4DIP	1,587491327	0,03650513
FGF12	1,69959574	0,03658266
SMIM3	4,001910433	0,0367096
TMTC3	1,037243829	0,03684921
TWF1	1,12245448	0,03698468
SRGAP1	1,530644889	0,03702657
UHRF1BP1L	1,069659502	0,03718902
CYTL1	2,828218599	0,0372651
HERC6	1,161936529	0,03728497
ME3	2,440681841	0,03731484
CA12	3,396761802	0,03762308
VIM	1,440363953	0,03764679
B4GALT7	1,174018649	0,0377569
KLC1	1,143147862	0,0378015
PACRGL	1,110022787	0,03793843
FAM84B	3,848295942	0,03803035
IGDCC4	2,518961925	0,03804065
GPC6	1,610751182	0,03821239
PHLDB2	7,114783447	0,03822692
ARMT1	1,003329801	0,03837501
NMB	1,795222765	0,03850036
STK17A	1,6652129	0,03889835
STARD4	1,120127028	0,03898122
MAP1A	1,981345466	0,03900397
ANG	1,87868381	0,0390426
IFT46	1,21176101	0,03907149
RAPH1	1,336215597	0,03920841
OAS3	1,60866856	0,03932152
SHC2	1,521465897	0,03942679

HAUS6	1,006191213	0,03953097
SNX6	1,028569152	0,03961461
SSBP3	1,163027762	0,03974112
HIF1A	2,389977558	0,03981223
RDH10	1,486845546	0,03988596
SAMD9	1,606104246	0,04007321
PML	1,599884455	0,0401117
ITGAV	1,076864741	0,04020982
NPR2	2,362614758	0,04035631
GLIS3	3,215151042	0,04054408
HOXD11	2,792641897	0,04065354
ITGA5	3,406115632	0,0407321
BCL2L1	2,088305758	0,04080103
ATF3	1,286443029	0,04080222
AAK1	1,291389437	0,04090581
FRMD4A	2,452497717	0,04105678
CH17-140K24	2,755756857	0,04109601
ZNF226	1,21075044	0,04110315
CLU	3,472446366	0,04114953
SEMA6D	2,508371582	0,041265
ACOX2	2,732282072	0,04143365
ZNF821	1,125043403	0,04149339
SMOX	1,680589336	0,04167728
SEMA3A	1,713764606	0,04175629
KIF7	1,038745081	0,04184928
DPF3	5,005673362	0,04185666
RTN1	6,693795052	0,04200836
MEIS1	3,031290934	0,04213854
LTBP3	1,115350645	0,04217216
HCG4P5	2,659394881	0,04221584
CST3	1,391750437	0,04222442
ZNF569	1,175206041	0,04227276
POU3F2	1,42565214	0,04232956
TRAF3IP2-AS1	1,20722164	0,04255215
THBS3	1,499815657	0,04265354
RGMB	2,017392153	0,04283434
RP11-161H23	1,561356517	0,04318459
KCNJ10	2,289774254	0,04326342
FJX1	2,722988919	0,04380717
LOXL2	2,297057188	0,044092
FZD1	4,259045552	0,04417788
SLC2A3	1,563370385	0,04438246
DHRS4L2	2,134286811	0,04444468
TENM3	2,73554722	0,04445309
ABCA3	1,344497124	0,04451035
CHPF2	1,292565789	0,04454957
SLC35G2	2,399902221	0,04492035
SLC38A1	2,25426942	0,04499006
IQGAP1	1,299991835	0,04508563
WASH7P	1,085932805	0,04512754

NPTX1	5,978421261	0,04521007
NACAD	2,218066984	0,04521285
RTN3	1,203254386	0,04523974
EMP1	2,854251852	0,04527668
BVES	2,078421383	0,04537256
CDO1	3,512817476	0,0454191
CPQ	1,742921426	0,04542994
IDUA	1,232033829	0,04568925
SOX4	1,103244668	0,04569358
NRP2	4,027472871	0,04571829
SPRY1	4,141595059	0,04577393
CDH24	3,245229595	0,04578207
PCDH1	2,95210859	0,04583178
NDP	3,146408754	0,0458401
RBPMS	1,625065563	0,0459066
HIPK3	1,181219471	0,04619035
VASN	4,10194351	0,04627265
ADAM19	2,458869729	0,04637312
PPP2R5B	1,237188511	0,04656866
ZNF677	1,220885659	0,04661774
EPB41L5	1,006098842	0,0466922
TCN2	7,067244145	0,04670959
HOXA6	1,041461698	0,04682816
ARMCX2	2,092627437	0,04703225
SNTA1	1,467653518	0,04710048
CSRNP1	1,826201687	0,04718195
AFF1	1,040144355	0,04721129
TGFB3	3,890417492	0,04745013
PSME2	1,100061277	0,04745805
SLC9A1	1,22364458	0,0474591
CNN3	1,154202559	0,04766229
ASPH	1,534421009	0,04772293
NLGN1	1,349883101	0,04774949
FAM89A	1,207108527	0,04777733
NMI	1,375011205	0,04778563
ARHGAP11A	1,008730984	0,04786721
PTPRZ1	1,251893427	0,04795475
LPP	1,06727582	0,04846331
SULF2	3,688913465	0,04854182
APLP2	3,705629366	0,0485705
FAM126A	1,162729242	0,04866551
FAT1	1,362586222	0,0487834
SYT11	2,332945047	0,04880226
SERPINH1	3,712887162	0,04880263
ST3GAL1	1,956646287	0,04883156
C3orf80	1,963349286	0,04887667
SNX9	1,201591482	0,04954924
ZNF853	1,728673526	0,04957276
MLLT3	1,49833706	0,04964392
HDAC9	4,395932555	0,04967727

RBPJ	1,462827474	0,0498896
ACSL4	2,046420168	0,04989622
