# Supplementary material for: PEA3 Transcription Factors, Role in Invasion, Proliferation and Radioresistance of Glioblastoma Stem Cells
Source: J Cell Mol Med. 2025 Apr 24;29(8):e70533. doi: 10.1111/jcmm.70533 (PMC12022000; doi:10.1111/jcmm.70533)
Supplement: Supplementary file 3 — Table S2. Genes positively correlated to high expression of ETV1 compared to low expression obtained from TCGA‐GBM database analysis. [file JCMM-29-e70533-s003.pdf]

Supplementary table 2:
Genes positively correlated to high expression of ETV1 compared to low expression obtained from TCGA-GBM database analysis (fold change cutoff 2.0 and a p-value <0.05).

geneID	logFC (ETV1 high/low)	P.Value
ETV1	2,672	1,03E-119
SOX11	2,145	8,32E-20
FABP7	2,069	1,06E-13
CSPG5	1,927	1,93E-23
BCAN	1,913	1,86E-32
NCAN	1,857	2,59E-16
TRIB2	1,851	1,30E-35
PTPRZ1	1,785	1,98E-12
PMP2	1,767	1,20E-09
ASCL1	1,746	2,23E-21
BCHE	1,736	1,97E-16
KCNIP1	1,702	2,79E-22
PDE4B	1,673	2,67E-32
GPM6A	1,669	4,69E-11
GRIA2	1,655	1,83E-12
C1orf61	1,645	7,15E-15
NES	1,605	8,12E-14
TTYH1	1,583	1,45E-14
SOX2	1,554	1,27E-31
SCRG1	1,553	4,10E-10
CCND2	1,54	5,92E-17
DLL3	1,513	9,97E-14
NKX2.2	1,501	3,39E-13
PTN	1,49	1,27E-13
TRIM9	1,49	3,82E-13
OLIG2	1,454	9,54E-26
MAP2	1,449	3,48E-22
TMSB15A	1,436	5,947E-06
ZEB1	1,434	5,25E-18
EDNRB	1,426	7,38E-14
LPHN3	1,416	4,73E-17
GAP43	1,413	2,54E-10
LRRTM2	1,412	1,50E-13
IGFBP2	1,405	2,04E-11
INSM1	1,4	5,80E-11
ASTN1	1,383	6,96E-15
SEMA5A	1,377	1,09E-18
FHL1	1,368	1,01E-19
FYN	1,362	1,84E-19
TUBB2B	1,35	8,85E-11
TIMP4	1,34	2,54E-09
PHLDA1	1,338	2,10E-25
NDRG2	1,332	3,98E-12
GPM6B	1,327	3,85E-09
BAALC	1,318	6,96E-09

NOVA1	1,317	1,83E-22
DCX	1,311	3,649E-06
EGFR	1,309	1,76E-07
ARC	1,305	9,20E-14
RCAN1	1,29	1,22E-11
FXYD6	1,273	4,09E-11
EXOC5	1,263	4,16E-39
HIP1	1,263	5,70E-31
RASSF2	1,246	3,90E-15
TMED2	1,243	1,96E-15
ZIC1	1,242	3,08E-10
CORO2B	1,233	2,84E-13
WSCD1	1,231	1,48E-18
CREB5	1,227	2,15E-20
NLGN1	1,224	9,91E-16
KCND2	1,221	3,11E-09
FAM70A	1,218	7,36E-10
SALL1	1,214	2,00E-17
FAM5C	1,214	1,46E-09
CRMP1	1,186	2,08E-11
LPL	1,174	1,30E-08
GRIA3	1,173	9,86E-18
KLHDC8A	1,173	1,57E-13
ZNF227	1,169	8,00E-35
CLIC4	1,166	2,65E-19
KCNJ16	1,161	2,345E-06
GPR56	1,155	5,87E-25
KIF15	1,15	3,19E-17
RHOBTB3	1,15	8,70E-20
TSPAN7	1,147	7,83E-10
PBK	1,144	3,85E-09
S100B	1,142	6,27E-07
SNTG1	1,137	3,70E-10
TOP2A	1,135	4,61E-09
SOX9	1,134	1,79E-13
GATM	1,132	1,09E-10
DPP6	1,131	1,27E-08
GLDC	1,131	6,18E-13
LRRN3	1,129	9,36E-10
SMARCA4	1,127	7,04E-23
TPX2	1,127	1,54E-13
AQP4	1,125	1,13E-07
NLGN4X	1,118	3,60E-11
ELOVL2	1,115	3,91E-09
CLIP2	1,11	7,09E-15
CRB1	1,11	1,03E-17
TRIO	1,106	3,57E-19
TMEM100	1,097	3,10E-07
COL9A3	1,096	4,87E-08
CRYAB	1,096	1,76E-07

FAM131B	1,093	2,92E-12
ATP1B2	1,092	1,05E-07
AGT	1,086	2,844E-06
KCNQ2	1,081	6,47E-15
SYT11	1,075	5,26E-12
SLC24A3	1,074	2,35E-12
CNTN1	1,066	8,48E-11
SCN3A	1,064	1,074E-06
ATAT1	1,062	7,78E-15
LIMA1	1,057	5,15E-20
ANGPT2	1,055	1,06E-11
FAM64A	1,054	1,20E-13
ABAT	1,052	3,01E-08
CTNND2	1,05	5,57E-09
MSH6	1,049	1,60E-21
FZD3	1,046	1,92E-17
SCARB2	1,041	2,41E-27
CALCRL	1,034	2,54E-14
PRKDC	1,028	8,38E-22
STK17A	1,026	4,76E-19
ID4	1,026	1,94E-07
FOXG1	1,023	7,31E-13
ANK2	1,02	6,89E-14
LPPR1	1,019	0,0006205
ZNF711	1,016	4,70E-26
SRSF1	1,016	8,54E-29
DCTN4	1,012	2,12E-40
CDH2	1,01	1,21E-17
SMC4	1,005	2,65E-12
OMG	1,002	0,00002131
