# Supplementary material for: PEA3 Transcription Factors, Role in Invasion, Proliferation and Radioresistance of Glioblastoma Stem Cells
Source: J Cell Mol Med. 2025 Apr 24;29(8):e70533. doi: 10.1111/jcmm.70533 (PMC12022000; doi:10.1111/jcmm.70533)
Supplement: Supplementary file 4 — Table S3. Genes positively correlated to high expression of ETV1 compared to low expression obtained from Rembrandt database analysis. [file JCMM-29-e70533-s002.pdf]

Supplementary table 3:
Genes positively correlated to high expression of ETV1 compared to low expression obtained from Rembrandt-GBM database analysis (fold change cutoff 2.0 and a p-value <0.05).

	logFC	P.Value
SOX11	2,736	4,65E-48
LHFPL3	2,692	2,80E-39
ETV1	2,462	2,18E-149
OLIG2	2,339	7,11E-51
BCHE	2,332	1,43E-43
TMEM100	2,265	8,56E-24
UHRF1	2,225	3,38E-46
LRRN1	2,213	1,41E-39
SOX8	2,161	2,89E-33
TMSB15A	2,064	4,93E-26
LPL	2,062	2,59E-30
DCX	2,027	1,06E-28
TIMP4	1,971	1,63E-31
PHLDA1	1,964	5,75E-45
NXPH1	1,934	3,51E-24
ASCL1	1,891	2,85E-34
KLRC3	1,881	1,58E-25
CALCRL	1,804	2,56E-35
SOX4	1,8	4,01E-35
BMP2	1,75	9,35E-23
LIMA1	1,744	4,36E-63
ZEB1	1,738	1,16E-63
PBK	1,736	4,07E-17
SHD	1,726	9,49E-27
VCAN	1,691	2,37E-31
TRIB2	1,674	5,12E-46
GLCCI1	1,669	5,95E-53
MTHFD2	1,625	3,18E-23
EDNRB	1,6	8,51E-37
OLIG1	1,589	8,57E-17
LOC389831	1,579	9,91E-41
NKAIN4	1,568	1,67E-25
LOC645323	1,546	1,43E-36
PCDHGC3	1,52	1,18E-65
CCND2	1,496	1,61E-40
MAML2	1,475	3,06E-57
C1orf61	1,459	1,11E-51
BEST3	1,454	1,06E-29
CRISPLD1	1,448	6,75E-18
FAM5C	1,443	2,23E-22
CASK	1,441	1,17E-64
KCND2	1,44	2,47E-15
GPR17	1,428	3,11E-11
LMNB1	1,415	8,51E-26
ID4	1,41	1,41E-27

SETD5	1,405	9,65E-70
VANGL2	1,39	1,59E-31
SALL3	1,385	1,41E-31
ZNF711	1,38	2,57E-35
HEY1	1,36	2,34E-34
TOX3	1,355	2,13E-13
DSCAM	1,346	1,05E-29
CPXM1	1,346	2,75E-27
SOX6	1,344	7,25E-45
TOP2A	1,341	1,00E-12
CSPG5	1,333	1,04E-26
FERMT1	1,305	1,12E-24
GRIA4	1,295	1,87E-21
PTPRZ1	1,294	2,25E-33
SULF2	1,279	2,15E-21
EZH2	1,278	1,62E-15
CACNG4	1,273	2,05E-36
DHX9	1,265	3,04E-36
MARCKS	1,26	9,78E-49
SMOC1	1,241	1,31E-19
NOVA1	1,238	4,27E-47
DLL3	1,237	2,65E-43
RNF180	1,233	3,92E-24
ZNF22	1,228	8,13E-60
PDE4B	1,228	8,18E-31
C3orf70	1,22	7,15E-29
NCAN	1,218	8,74E-16
NFIB	1,217	6,42E-35
NUSAP1	1,214	3,07E-15
RRM2	1,211	9,92E-11
sept-02	1,206	9,52E-43
NMB	1,205	2,96E-18
HIP1	1,204	5,04E-54
KCNIP1	1,202	1,81E-24
LOC650392	1,198	3,20E-15
NLGN1	1,192	3,50E-31
SOX2	1,192	2,53E-35
PCDHB10	1,183	9,58E-28
CHST9	1,179	1,88E-13
NKX2.2	1,178	6,04E-20
FAM110B	1,178	1,91E-29
FREM2	1,177	4,39E-12
ZNF300	1,175	9,23E-37
LOC643763	1,163	3,32E-13
NUF2	1,162	2,36E-11
ANGPTL2	1,148	2,71E-21
DDX42	1,143	1,46E-25
MMP16	1,139	9,50E-49
GLDC	1,128	1,03E-32
C1orf106	1,127	1,22E-39

TP53	1,124	4,27E-29
WSCD1	1,123	3,38E-33
UNQ6228	1,119	2,91E-21
LYPD1	1,117	1,02E-19
GALNT13	1,111	2,24E-11
KIF11	1,11	5,08E-16
SEMA5A	1,109	1,12E-28
GPR56	1,108	1,71E-39
ID3	1,104	1,80E-14
SLC39A6	1,101	2,42E-47
KIAA1211	1,096	2,98E-25
DTL	1,092	1,62E-13
DPYSL3	1,086	1,39E-26
FAM70A	1,082	2,64E-11
PRRX1	1,075	1,53E-19
ADAM9	1,071	1,32E-18
TMEM97	1,07	4,45E-30
LAPTM4B	1,063	3,14E-44
CRB1	1,061	1,41E-20
FYN	1,061	2,82E-44
ODC1	1,06	3,13E-30
MYT1	1,059	7,80E-35
BTBD17	1,054	8,49E-33
CHST11	1,05	4,56E-43
DBF4	1,048	6,26E-18
CDK1	1,045	2,31E-15
TSPAN11	1,042	1,46E-33
KIF15	1,042	1,26E-15
TNFRSF21	1,032	2,26E-26
FGFBP3	1,032	3,09E-25
ACLY	1,031	6,49E-35
LDLRAD3	1,03	2,53E-28
STK17A	1,029	5,18E-30
TMEM133	1,029	9,45E-28
EIF4A2	1,025	1,58E-21
FAM181B	1,023	1,21E-42
ZBTB20	1,022	6,96E-29
SCD5	1,02	2,68E-44
RFTN2	1,019	9,89E-27
CKS2	1,019	5,67E-10
REPIN1	1,018	6,09E-35
MAD2L1	1,016	2,33E-16
FBXO5	1,012	1,30E-21
MBTD1	1,01	1,33E-46
NONO	1,01	1,96E-49
SCG3	1,008	1,20E-07
MAP2	1,007	3,15E-21
ZNF462	1,006	4,46E-35
FJX1	1,005	9,49E-26
HAPLN1	1,005	8,18E-13

KCNQ2	1,002	3,06E-21
PAICS	1,001	4,90E-27
